# A chronic *Pseudomonas aeruginosa* mouse lung infection modeling the mucus obstruction, lung function, and inflammation of human cystic fibrosis

**DOI:** 10.1128/iai.00230-25

**Published:** 2025-06-13

**Authors:** Mylene Vaillancourt, Sheryl E. Fernandes, Diane Aguilar, Anna Clara Milesi Galdino, Peter Jorth

**Affiliations:** 1 Department of Pathology and Laboratory Medicine, Cedars-Sinai Medical Center 22494, Los Angeles, California, USA; 2 Department of Biomedical Sciences, Cedars-Sinai Medical Center 22494, Los Angeles, California, USA; 3 Department of Medicine, Cedars-Sinai Medical Center 22494, Los Angeles, California, USA; Universite de Geneve, Genève, Switzerland

**Keywords:** cystic fibrosis, *Pseudomonas aeruginosa*, animal models, inflammation, lung infection

## Abstract

Mouse models of cystic fibrosis (CF) have been used to study chronic lung infections; however, these models have lacked the airway mucus that defines human CF pathophysiology and required the use of mucoid *Pseudomonas aeruginosa*. Alternative models have used either transgenic *Scnn1b-Tg* mice overexpressing a lung epithelial sodium channel to mimic the mucus-rich CF lung environment, synthetic CF sputum medium (SCFM2) to induce bacterial phenotypes consistent with human CF, or agar beads to promote chronic infections by non-mucoid *P. aeruginosa*. Here, we combined these alternative models and established a chronic *P. aeruginosa* lung infection model using SCFM2 agar beads and *Scnn1b-*Tg mice (SCFM2-*Scnn1b-*Tg) to recapitulate nutrient and mucus characteristics of the human CF lung environment and test the effects of chronic infections on bacterial burden, lung function, and the immune response. Using wild-type SCFM2-C57BL/6 mice as controls, SCFM2-*Scnn1b-*Tg mice failed to clear bacterial infections, and lung function measurements showed that infected SCFM2-*Scnn1b-*Tg mice had decreased inspiratory capacity and compliance, elevated airway resistance, and significantly reduced forced expiratory volumes. Flow cytometry and cytokine arrays showed that, like people with CF, SCFM2-*Scnn1b-*Tg mice developed inflammation characterized by neutrophil and eosinophil infiltration and Th2 lymphocytic cytokine responses. Chronically infected SCFM2-*Scnn1b-*Tg mice developed an exacerbated mix of innate and Th1, Th2, and Th17-mediated inflammation, causing higher lung cellular damage and elevated numbers of unusual Siglec F^+^ neutrophils. SCFM2-*Scnn1b-*Tg mice will be useful for investigating bacterial pathogenesis by non-mucoid *P. aeruginosa*, including treatments and the roles of Siglec F^+^ neutrophils in CF inflammation.

## INTRODUCTION

*Pseudomonas aeruginosa* is a gram-negative opportunistic bacterium responsible for persistent lung infections in people with muco-obstructive and chronic inflammation like cystic fibrosis (CF). Although primary *P. aeruginosa* infection does not immediately decrease lung function in most people with CF (pwCF) (1), adaptation and changes in *P. aeruginosa* virulence and antibiotic resistance during chronic infections are thought to be the most common cause of pulmonary exacerbations (2). Pulmonary exacerbations caused by bacterial infections are characterized by increased mucus production and an amplified inflammatory response leading to irreversible airway damage and a decrease in respiratory spirometry (2, 3). Studying bacterial interactions with the host environment has been challenging since *P. aeruginosa* modulates its gene expression in response to environmental nutrients and stress conditions, making *in vitro* characterization poorly relevant to *in vivo* infections (4, 5). To overcome this issue, synthetic CF sputum-mimicking medium (SCFM2) was developed, and *P. aeruginosa* grown in SCFM2 has genetic fitness determinants and gene expression profiles that mirror bacteria grown in sputum collected from pwCF (6–8). However, even with SCFM2, *in vitro* studies lack crucial host factors mediating host-pathogen interactions, including the highly inflammatory and oxidative environment produced by immune cells.

Since the discovery of the CF transmembrane conductance regulator (*CFTR*) gene 35 years ago, multiple *in vivo* murine models of chronic *P. aeruginosa* infections have been developed (9–11). To establish chronic *P. aeruginosa* infections in mice, different strategies have included growing the strains in aggregates or adding alginate to promote biofilm-like phenotypes (12–14), using fibrinogen plug models (15, 16), or embedding bacteria in agar beads (17–20). Using these strategies, most studies were successful in establishing chronic infections in mice. However, these models do not recapitulate CF infections as they utilized healthy mice lacking key pathological lung characteristics seen in human CF disease, including dense airway mucus and immune cell infiltration (13, 15, 18, 20, 21). CFTR mutant mice showed higher sensitivity to *P. aeruginosa* infections and developed higher inflammation compared to WT counterparts during chronic infections (17, 19). However, they did not spontaneously develop mucus plugs and complex inflammation underlying CF disease. Notably, the inflammatory response of these models was primarily neutrophilic, while human CF lung inflammation also has eosinophilia and lymphocytosis (22–24). In an attempt to improve the CF mouse model, CF mice with S489X *CFTR* mutations were infected with a mucoid clinical isolate of *P. aeruginosa* embedded in tryptic soy broth agarose beads. In this agar bead-CF mouse model, CF mice suffered higher mortality than normal mice, had higher inflammation, and experienced greater weight loss, but the CF mice did not have higher bacterial burdens and also lacked the mucus seen in most pwCF (25). While *CFTR* mutant mice may be ideal for studying direct interactions between *P. aeruginosa* and CFTR (26, 27), the lack of mucus plugging allows bacteria to make contact with the apical surface of airway epithelial cells, and these mice may not be ideal models to study the effects of obstructive pulmonary disease on bacterial physiology or the effects of long-term *P. aeruginosa-*neutrophil interactions. Another limitation to the CF mouse chronic infection model is that it depends on the use of mucoid *P. aeruginosa*. While mucoid *P. aeruginosa* with *mucA* mutations are commonly isolated from many pwCF and are associated with poor outcomes, mucoid conversion typically occurs after 10 years of infection dominated by non-mucoid isolates (28, 29). Research from our group and others has linked non-*mucA* mutations to poor outcomes, pathoadaptation, and antibiotic resistance (5, 30–33); therefore, it will be useful to have an *in vivo* model to study mutations in non-mucoid genetic backgrounds.

*Scnn1b* transgenic (*Scnn1b*-Tg) mice overexpress the βENaC epithelium sodium channel in their lungs, causing CF-like lung pathology including mucus accumulation and neutrophil infiltration (34, 35). These mice were described to spontaneously develop juvenile asthmatic inflammation that partially resolved in early adulthood (36). Furthermore, *Scnn1b*-Tg mice were more sensitive to infection and developed higher inflammatory responses to *P. aeruginosa* infection (14, 15), making them a compelling model to study chronic bacterial infections. However, the lung nutrient composition of *Scnn1b-Tg* mice is unknown, which has limited their utility as models for CF. The synthetic CF sputum medium (SCFM2) was used recently to pre-culture *P. aeruginosa* prior to acute lung infections of WT C57BL/6J mice and transcriptomic analyses of bacteria in infected mice revealed that SCFM2 promoted improved CF gene expression phenotypes in the infecting *P. aeruginosa* compared to bacteria pre-grown on Pseudomonas Isolation Agar (37). Yet, the authors acknowledged several limitations of this SCFM2 model and suggested that the *Scnn1b*-Tg mouse model could further recapitulate CF disease physiology, which we test here. Neither the cellular immune responses to infections nor the effects on lung respiratory mechanics have been well characterized in either *Scnn1b*-Tg mice or SCFM2-C57BL/6J mouse infections.

Here, we sought to overcome the limitations of previous murine CF chronic *P. aeruginosa* infection models. First, we used *Scnn1b*-Tg mice to recapitulate the underlying inflammation and obstructive lung pathology seen in human CF disease. Next, we used agar beads to promote biofilm aggregate formation and promote chronic infection with non-mucoid *P. aeruginosa* PAO1. We chose to use strain PAO1 because it is a model non-mucoid strain that is closely related to many CF isolates, and PAO1 is the parent strain for an ordered transposon mutant library which could be useful in future studies (38–41). Third, we used SCFM2 agar to recapitulate the human CF nutrient environment and promote CF-like gene expression in *P. aeruginosa*. We hypothesize that SCFM2 combined with *Scnn1b*-Tg mice would promote chronic infection by non-mucoid *P. aeruginosa*, decrease lung function, and increase innate immune responses to infection relative to WT mice. To test this, SCFM2-*Scnn1b*-Tg mice were infected with PAO1 and compared to SCFM2-C57BL/6 wild-type (WT) littermate infected mice, and we measured the effects on bacterial clearance, lung function, and immune responses to infection, using uninfected mice inoculated with sterile SCFM2 agar beads as controls.

## RESULTS

### Bacterial clearance is impaired in SCFM2-*Scnn1b*-Tg mouse model

**Fig 1:**
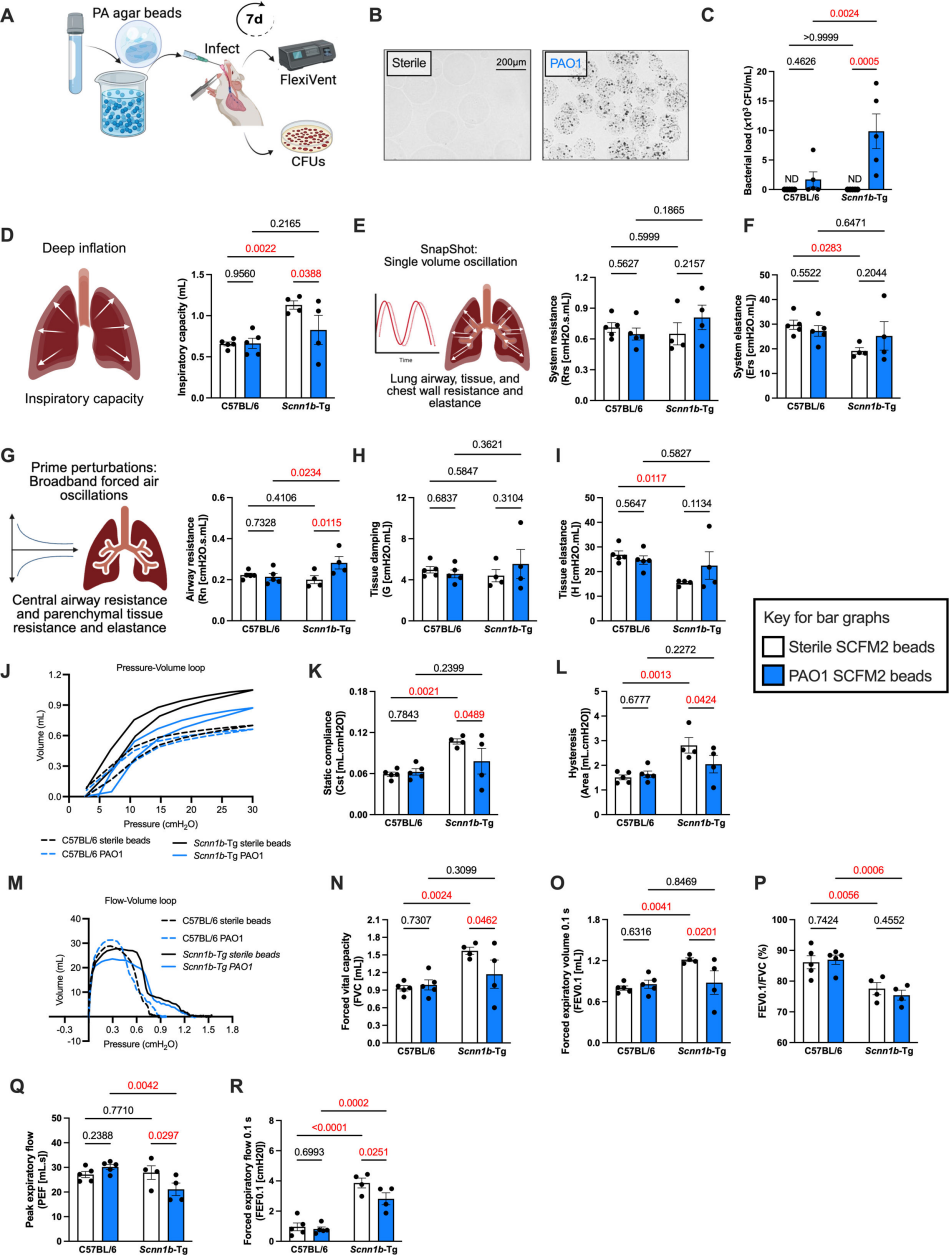
Bacterial clearance is impaired in SCFM2-*Scnn1b*-Tg mice and increases airway resistance during chronic infection. (**A**) WT C57BL/6 or *Scnn1b*-Tg mice were intratracheally inoculated with sterile or 1 × 10^6^ CFU PAO1-laden SCFM2-agar beads. (**B**) Representative microscopic images of sterile and PAO1-laden SCFM2-agar beads. (**C**) Bacterial load in mouse lungs 7 days post-infection. Lung bacterial load was determined by CFU/mL. (**D–R**) Lung function measurements were obtained using the flexiVent (SCIREQ). (**D**) Inspiratory capacity using a deep inflation technique. (**E and F**) System resistance (**E**) and elastance (**F**) parameters were acquired by the single-frequency forced oscillation maneuver. (**G–J**) Airway resistance (**G**), tissue resistance (damping) (**H**), elastance (**I**), and hysteresivity (**J**) were obtained from the low-frequency forced oscillation technique. (**J**) Representative graph of pressure-volume loop. (**L**) Static compliance (**K**) and hysteresis (**L**) were obtained by a pressure-volume loop. Increased static compliance and hysteresis reflect emphysema and alveolar damage in uninfected *Scnn1b*-Tg mice compared to WT mice. (**M**) Representative graph of the forced expiratory volume perturbation. Spirometry measurements from flow-volume loop: FVC (**N**), FEV0.1 (**O**), FEV0.1/FVC (**P**), PEF (**Q**), and FEF0.1 (**R**). (**C–R**) *n* = 4–5 mice/group, *P* values indicate two-way ANOVA analyses, red *P* values indicate *P* < 0.05, white bars indicate sterile SCFM2 agar bead controls, and blue bars indicate SCFM2 agar beads with PAO1.

The primary goal of our study was to determine if we could establish a chronic infection with non-mucoid *P. aeruginosa* using SCFM2 and *Scnn1b-*Tg mice. We embedded *P. aeruginosa* PAO1 in SCFM2 (7) agar beads (Fig. 1A and B). We intratracheally inoculated 1 × 10^6^ colony-forming units (CFUs) or sterile SCFM2 agar beads into *Scnn1b*-Tg mice or their WT C567BL/6 littermates. Following preparation, agar beads are always used within 1 week, and control experiments show that bacteria retain viability up to at least 2 weeks after preparation (Fig. S1). After 7 days of infection, the bacterial load was more than fivefold higher in SCFM2-*Scnn1b*-Tg infected mice compared to their WT SCFM2-C57BL/6 littermates (Fig. 1C). These data also showed that while some WT mice cleared the infections by 7 days, all SCFM2-*Scnn1b-*Tg mice remained infected (Fig. 1C). These results confirm that bacterial clearance is impaired in SCFM2-*Scnn1b*-Tg mice during chronic infection.

### Infected SCFM2-*Scnn1b*-Tg mice develop a mixed obstructive and restrictive lung disease

Changes in lung function are often used to determine the effectiveness of therapies in clinical trials for pwCF (42). *Scnn1b-*Tg mice have previously been shown to exhibit pulmonary dysfunction similar to pwCF (43); however, lung function has not been measured in response to *P. aeruginosa* infection in this model. While we hypothesized that lung function would decrease in *Scnn1b-*Tg mice infected with *P. aeruginosa*, the possibility remained that preexisting pulmonary dysfunction in *Scnn1b-*Tg mice would mask changes caused by *P. aeruginosa* infection. Therefore, we measured the effects of chronic non-mucoid *P. aeruginosa* PAO1 infection on pulmonary function to assess whether this model would be useful for measuring changes induced by infection.

To determine the effects of chronic infection on pulmonary function, it was essential to first determine lung function properties at baseline and use sterile agar bead controls to account for the potential effects of agar beads on lung function. We analyzed properties of the total lung system, including total lung inspiratory capacity, as well as resistance and elastance of the total lung system encompassing the conducting and peripheral airways, lung tissue, and chest walls using a flexiVent system (SCIREQ). At baseline and at 7 days post-inoculation with sterile bead controls, the deep inflation technique showed that *Scnn1b*-Tg mice had significantly higher inspiratory capacities compared to their WT littermates (Fig. S2A; Fig. 1D). The SnapShot technique introduces a single air volume oscillation into the lungs to measure the combined system resistance and elastance of the lung airways, lung tissue, and chest walls. *Scnn1b*-Tg mice had equivalent system resistance to WT mice (Fig. S2B; Fig. 1E) and lower system elastance than WT mice (Fig. S2C; Fig. 1F). Next, we asked whether changes in these total system properties could be explained by differences in the properties of the large conducting airways and parenchymal lung tissue of *Scnn1b*-Tg mice using the prime perturbations technique, which introduces broadband forced air oscillations into the lungs (Fig. 1G). No differences were observed in the baseline resistance of the large conducting airways (Fig. S2D) of the *Scnn1b*-Tg mice and WT mice or in the tissue damping (Fig. S2E) which reflects resistance in the parenchymal lung tissue, and these properties remained consistent with sterile bead inoculation (Fig. 1G and H). However, the parenchymal tissue elastance was significantly lower in the *Scnn1b*-Tg mice (Fig. S2F) and remained lower than WT 7 days after sterile bead inoculation (Fig. 1I). Analyses of pressure-volume (PV) curves (Fig. S2B) revealed higher hysteresis and static compliance (Fig. S2G and H) in *Scnn1b*-Tg mice at baseline and 7 days after sterile agar bead inoculation (Fig. 1J through L), confirming the presence of underlying emphysema and damage to alveoli in *Scnn1b*-Tg mice. Finally, consistent with previous research (43), we observed greater forced vital capacity (FVC; Fig. S2J) and forced expiratory volume in 0.1 s (FEV0.1; Fig. S2K), with reduced FEV0.1/FVC (Fig. S2L), no change in peak expiratory flow (PEF; Fig. S2M), but increased forced expiratory flow in 0.1 s (Fig. S2N and O) in *Scnn1b*-Tg mice relative to WT at baseline, and these differences remained consistent 7 days after sterile agar bead inoculation (Fig. 1M through R). Altogether, these findings suggest that *Scnn1b*-Tg mice have underlying emphysema like many pwCF, and inoculation of *Scnn1b*-Tg mice with sterile agar beads does not change these properties relative to WT C57BL/6 mice littermates.

Defining baseline pulmonary function in *Scnn1b*-Tg mice and assessing the effects of sterile agar bead controls enabled us to study the effects of chronic *P. aeruginosa* PAO1 infection on pulmonary function. Chronic infection significantly decreased the inspiratory capacity of *Scnn1b*-Tg mice (Fig. 1D) and increased central airway resistance (Fig. 1G). Both compliance and hysteresis were decreased during chronic infection, indicating restriction during inhalation (Fig. 1J through L). The negative pressure-driven forced expiration (NPFE) maneuver flow-volume curves showed a decrease in the FVC, FEV0.1, PEF, and FEF0.1 relative to sterile agar bead controls (Fig. 1M through R), consistent with an obstructive pathology following infection. Taken together, the lung mechanics confirmed an initial obstructive lung disease in *Scnn1b*-Tg mice and demonstrated the establishment of a mixed obstructive/restrictive pathology during chronic infection in the SCFM2-*Scnn1b*-Tg mice.

### Atypical neutrophils increase during infection of SCFM2-*Scnn1b*-Tg mice

**Fig 2:**
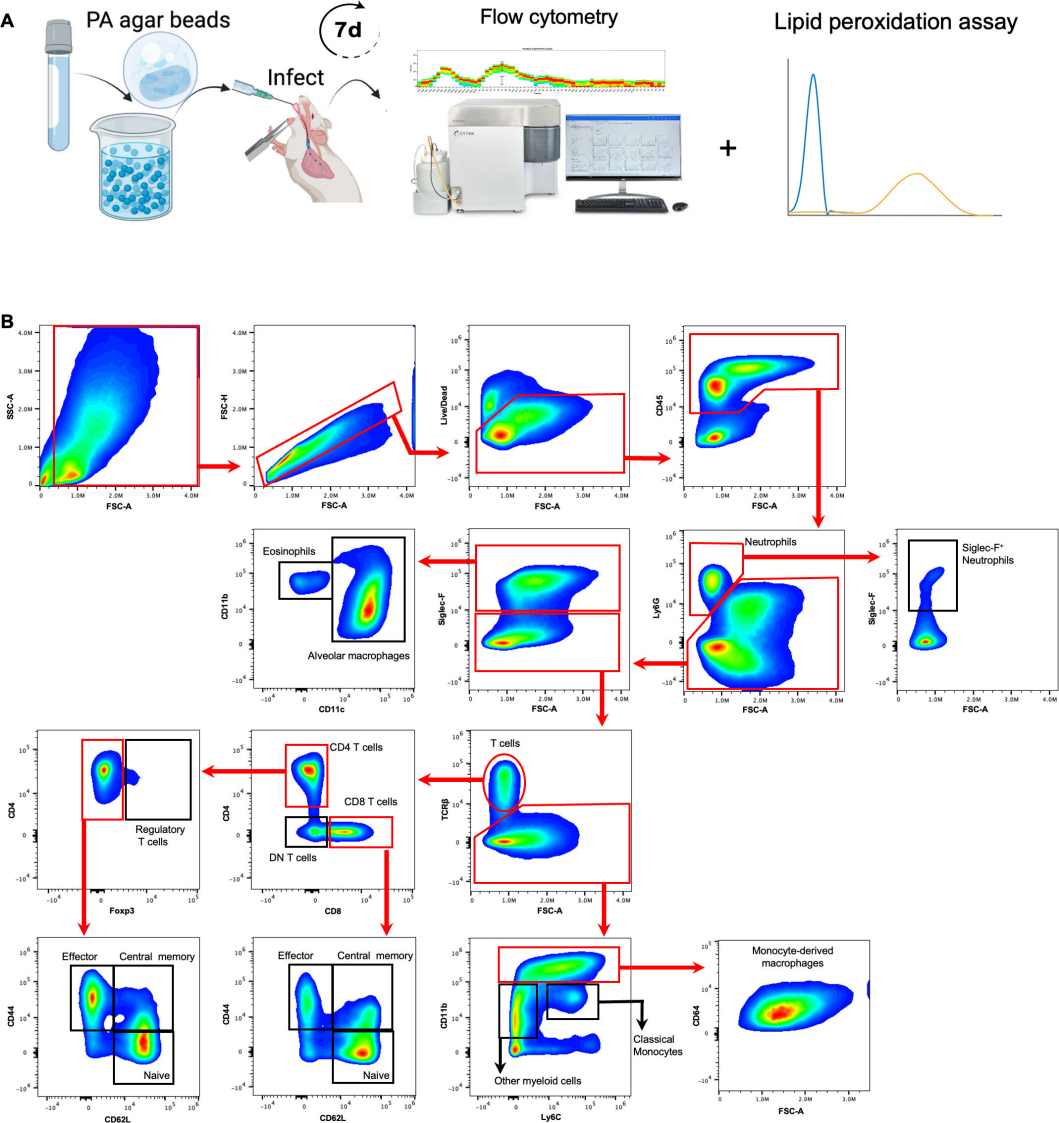
Flow cytometry strategy to study the immune response to chronic *P. aeruginosa* infection in WT SCFM2-C57BL/6 or SCFM2-*Scnn1b*-Tg mice. (**A**) WT C57BL/6 or *Scnn1b*-Tg mice were intratracheally inoculated with sterile or 1 × 10^6^ CFU PAO1-laden SCFM2-agar beads. (**B**) Gating strategy was used to identify immune cell response at day 7 post infection. Cells were isolated from enzymatically digested mouse lungs, and, after the exclusion of doublets and debris, live and immune cells were identified by LIVE/DEAD staining and CD45 staining. Neutrophils (Ly6G^+^) were isolated and gated for the Siglec F marker. Then, Ly6G^−^ and Siglec F^+^ cells were selected to differentiate alveolar macrophages (Siglec-F^+^ and CD11c^+^) and eosinophils (CD11b^+^, CD11c^−^, and Siglec-F^+^). T cells (TCRβ^+^) were then separated from the rest of Sigle F^−^ cells. CD4^+^ and CD8^+^ were separated from the double-negative (DN) subset. CD4^+^ and Foxp3^+^ cells were isolated, while Foxp3^−^ cells were separated by the CD44 and CD62L markers to identify naïve CD4^+^ T cells (TCRβ^+^, CD4^+^, CD44^−^, and CD62L^+^), effector CD4^+^ T cells (TCRβ^+^, CD4^+^, CD44^+^, and CD62L^−^), and central memory CD4^+^ T cells (TCRβ^+^, CD4^+^, CD44^+^, and CD62L^+^). CD8^+^ T cells were also separated with the same markers CD44 and CD62L. Finally, TCR^−^ cells were further separated using Ly6C and CD11b markers to identify monocyte-derived macrophages (CD11b^High^, Ly6C^+/−^, and CD64^+^), classical monocytes (CD11b^+^ and Ly6C^+^), and other myeloid-derived cells (CD11b^+^ and Ly6C^−^).

**Fig 3:**
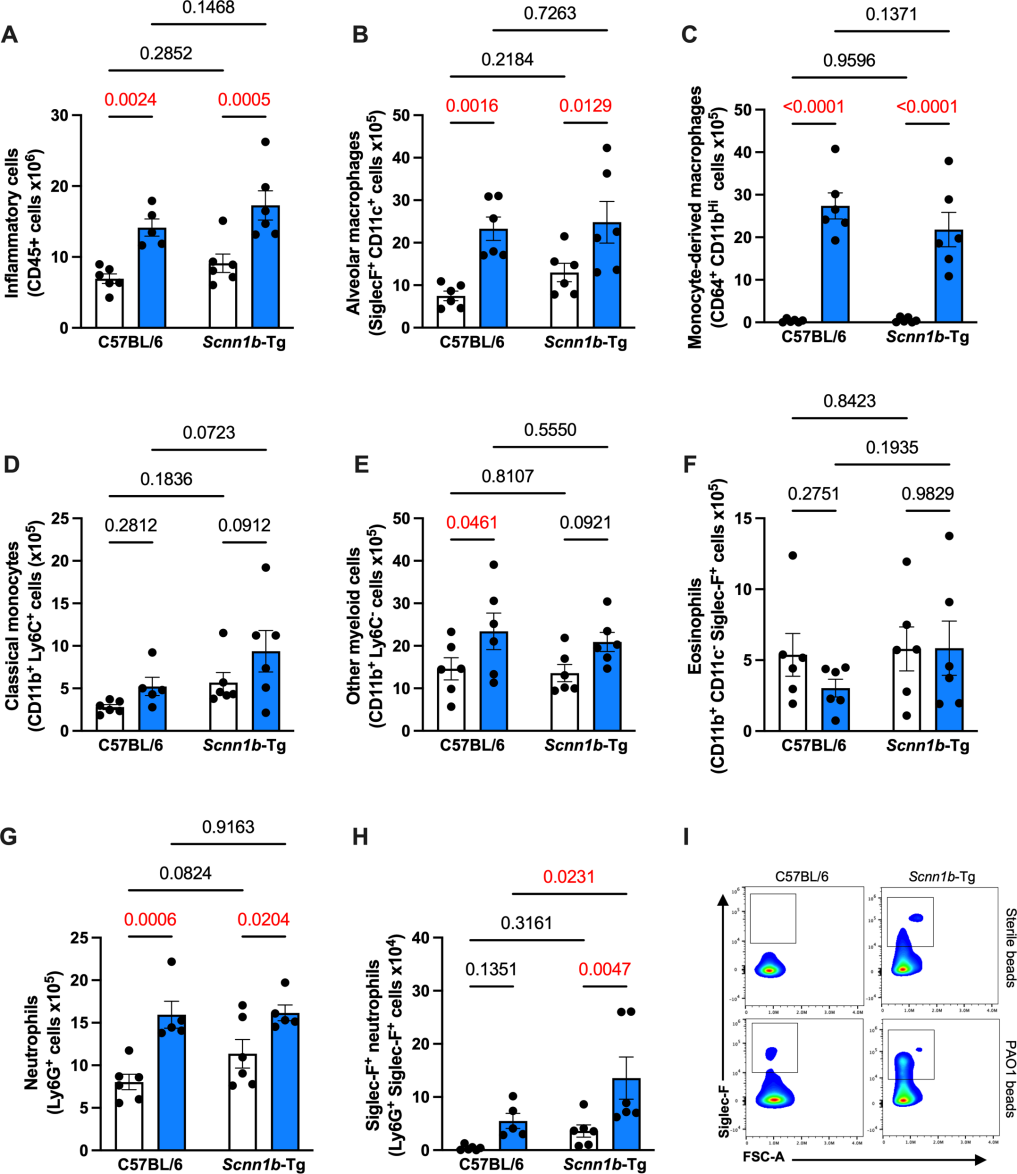
Infection of SCFM2-*Scnn1b*-Tg mice leads to an increase in atypical neutrophils. (**A**) Inflammatory cells were increased in both *Scnn1b*-Tg mice and their WT littermates. (**B–E**) Different innate cells were upregulated in both genotypes during chronic infection. (**B**) Alveolar macrophages. (**C**) Monocyte-derived macrophages. (**D**) Classical monocytes. (E) Other myeloid cells. (**F**) Eosinophils were not upregulated during chronic infection with *P. aeruginosa*. (**G**) Neutrophils were upregulated during chronic infection but not modulated by the genotype. (**H and I**) An atypical Siglec F^+^ neutrophil subset was upregulated in *Scnn1b*-Tg mice during chronic infection. For (A–H), white bars indicate sterile SCFM2 agar bead controls, blue bars indicate SCFM2 agar beads with PAO1, *n* = 6 mice/group, mean ± SEM, two-way ANOVA, *P* < 0.05 are highlighted in red.

*Scnn1b*-Tg mice were previously described to develop chronic airway inflammation characterized by increased macrophages, neutrophils, eosinophils, and lymphocytes (34, 35). We confirmed the presence of lung inflammation in the bronchoalveolar lavage (BAL) of uninfected *Scnn1b*-Tg mice (Fig. S3 and S4). To determine whether this underlying inflammation could affect the inflammatory response to bacterial infection, we infected *Scnn1b*-Tg mice or their WT littermates with PAO1-laden or sterile SCFM2 agar beads (Fig. 2A). At day 7 post-infection, we quantified total lung immune cell populations by spectral flow cytometry (Fig. 2B). As expected, total inflammatory cells were increased in infected mice of both genotypes (Fig. 3A). Alveolar and monocyte-derived macrophages were also increased in infected mice (Fig. 3B and C). Neither infection led to changes in classical monocytes (Fig. 3D), and the inflammation in WT mice was characterized by an increase in other CD11b^+^ myeloid cells (Fig. 3E). While eosinophils were higher in BAL of uninfected *Scnn1b*-Tg compared to WT mice (Fig. S2E), there was no difference in lung eosinophil numbers with sterile beads or during chronic infection (Fig. 3F). A surprising finding in infected SCMF2-*Scnn1b*-Tg mice was the upregulation of an atypical Siglec-F^+^ neutrophil subset (Fig. 3H and I), despite no difference in total neutrophils between the two mouse genotypes during infection (Fig. 3G). Taken together, these results show that SCFM2-*Scnn1b-*Tg mouse infections recruit macrophages and neutrophils to the lungs, with a marked increase of unusual Siglec-F^+^ neutrophils unique to the SCFM2-*Scnn1b-*Tg model system.

### Lymphocyte populations increase during SCMF2-*Scnn1b-*Tg infections

**Fig 4:**
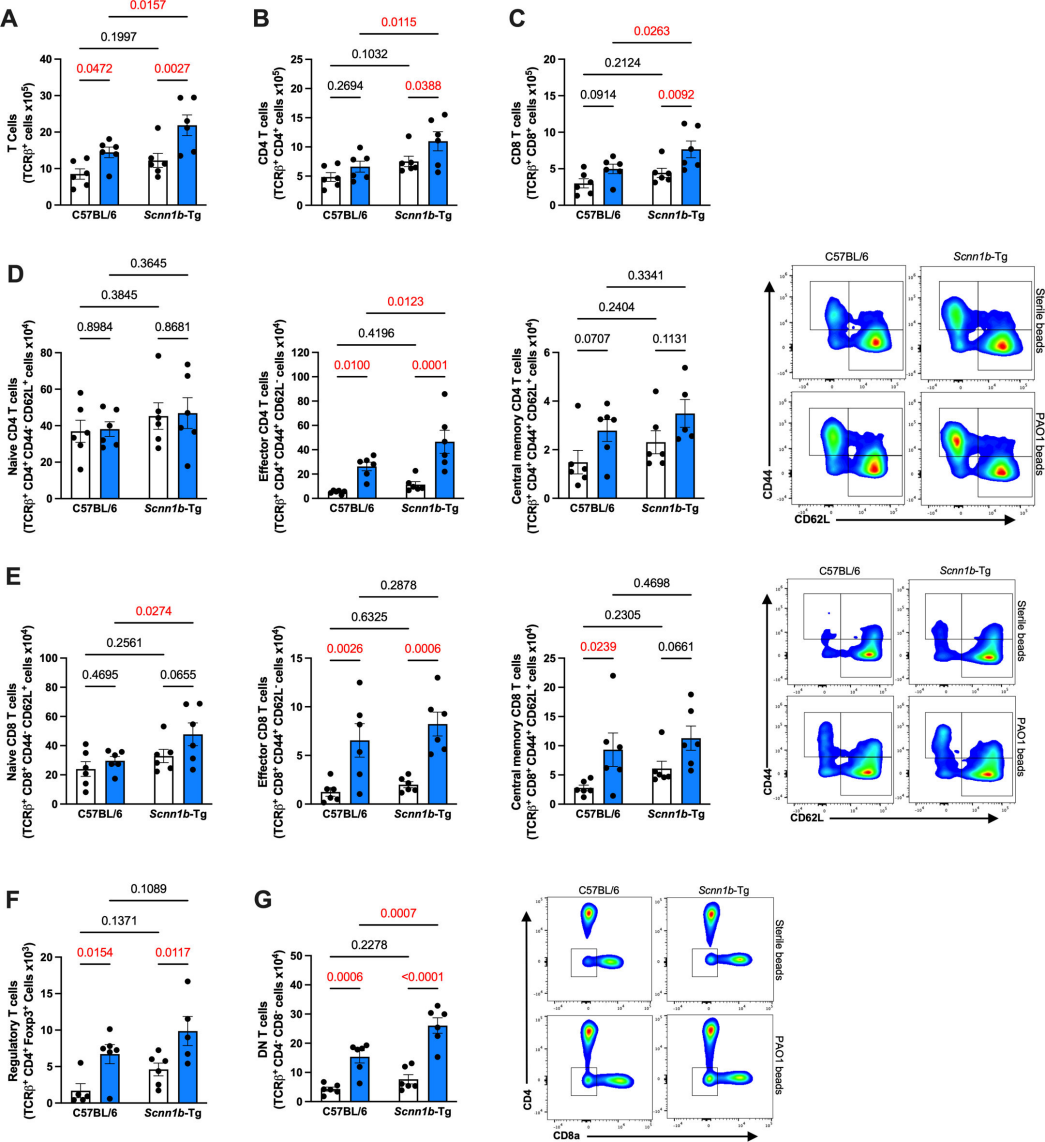
Effector T cells increase during *P. aeruginosa* infection in SCFM2-*Scnn1b*-Tg-mice. (**A**) Total T cells were significantly increased during chronic infection and even more in the *Scnn1b*-Tg mice. (**B and C**) This increase in T cells was explained by higher numbers of CD4^+^ (**B**) and CD8^+^ (**C**) T cells. (**D**) Activation state of CD4^+^ T cells. No difference was seen in naïve CD4^+^ T cells. During chronic infection, a significant upregulation of effector T cells was observed in both genotypes, and this increase was greater in *Scnn1b*-Tg mice compared to their WT littermates. A modest but non-significant increase was detected for central memory T cells in infected mice. (**E**) Activation state of CD8^+^ T cells. During chronic infection, a significant increase in naïve CD8^+^ T cells was observed in *Scnn1b*-Tg mice. Effector T cells were also increased in both genotypes. A modest increase of central memory CD8 was detected for both genotypes. (**F**) Regulatory T cells were also increased in all infected mice but not modulated by the genotype. (**G**) Double-negative (DN) cells were significantly increased in all infected mice and were significantly higher in *Scnn1b*-Tg mice compared to their WT littermates. For (A–G), white bars indicate sterile SCFM2 agar bead controls, blue bars indicate SCFM2 agar beads with PAO1, *n* = 6 mice/group, mean ± SEM, two-way ANOVA, *P* < 0.05 are highlighted in red.

To further characterize the inflammatory response of *Scnn1b*-Tg mice during chronic infection, we quantified different lymphocyte subtypes and their activation states. As expected during chronic infection, infiltrating T cells were present in the lung tissues of all infected mice (Fig. 4A). Infection significantly increased both CD4^+^ and CD8^+^ T cells in SCFM2-*Scnn1b*-Tg but not SCFM2-C57BL/6 mice (Fig. 4B and C). We then separated the cells on whether they were activated or naïve. In each subtype, a small proportion of cells were central memory T cells detected based on their positivity for both markers (44). Infected mice of both genotypes showed increased effector CD4^+^ T cells during chronic infection (Fig. 4D), but effector CD4^+^ T cells were also significantly higher in infected SCFM2-*Scnn1b*-Tg mice compared to WT SCFM2-C57BL/6 infected mice. No changes in naïve CD4^+^ T cells or central memory CD4^+^ T cells were observed during either infection (Fig. 4D). In contrast, naïve CD8^+^ T cells were higher in SCFM2-*Scnn1b*-Tg mice during infection compared to WT SCFM2-C57BL/6 infected mice (Fig. 4E). Effector CD8^+^ T cells increased during infection in both SCFM2-*Scnn1b*-Tg and WT mice (Fig. 4E), while central memory CD8^+^ T cells increased during infection of WT SCFM2-C57BL/6 infected mice only (Fig. 4E). Because regulatory T cells can modulate the immune response (45), we quantified them in the infected lungs of infected SCFM2-*Scnn1b*-Tg and SCFM2-C57BL/6 mice (Fig. 4F). Although regulatory T cells were more abundant in infected mice, there was no difference between the two mouse genotypes (Fig. 4F). Finally, we observed a significant increase in CD4^−^ CD8^−^ double-negative (DN) T cells in the infected lungs of infected SCFM2-*Scnn1b*-Tg (Fig. 4G).

### Infected SCFM2-*Scnn1b*-Tg mice increase innate inflammatory signaling

**Fig 5:**
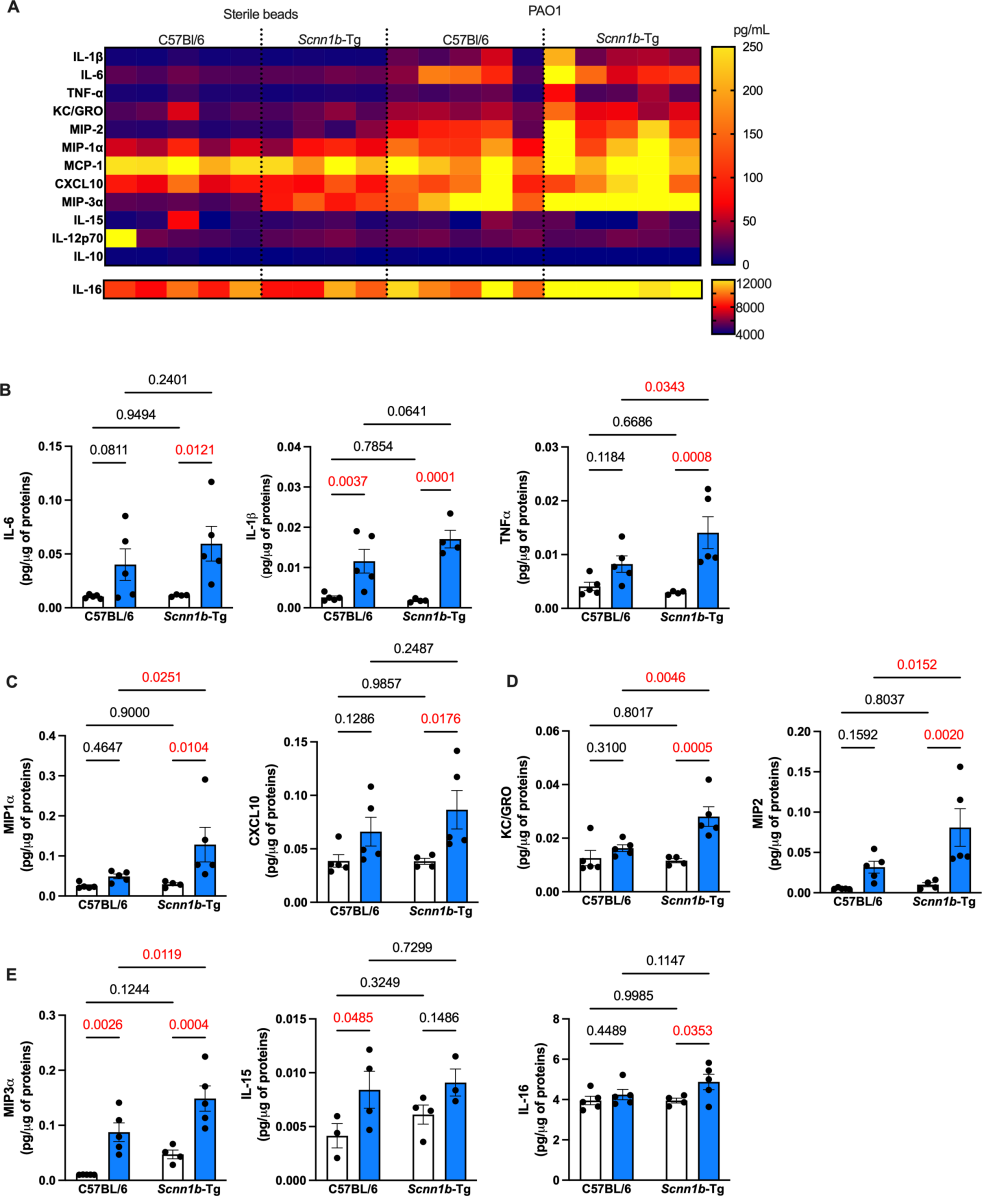
SCFM2-*Scnn1b*-Tg mice develop exacerbated innate inflammation during chronic infection. (**A**) Quantification (pg/μg of proteins) of pro- and anti-inflammatory cytokines and chemokines in whole lung lysates of chronically infected mice. (**B**) Inflammatory cytokines IL-6, IL-1β, and TNF-α were upregulated in all infected mice. IL-1β and TNF-α levels were higher in *Scnn1b*-Tg mice compared to their WT littermates. (**C**) Monocytes/macrophages chemoattractant MIP-1α and CXCL10 were significantly upregulated in infected *Scnn1b*-Tg mice. (**D**) Neutrophil chemoattractants were significantly upregulated in infected *Scnn1b*-Tg mice compared to their WT littermates. (**E**) Lymphocyte chemoattractant MIP-3α was upregulated in all infected mice but was higher in *Scnn1b*-Tg mice. IL-15 was increased in infected WT C57BL/6 mice only, while IL-16 was only upregulated in *Scnn1b*-Tg mice. For (B–E), white bars indicate sterile SCFM2 agar bead controls, blue bars indicate SCFM2 agar beads with PAO1, *n* = 4–5 mice/group, *P < 0.05, **P < 0.01, and ***P < 0.001. See Table S1 for statistical tests used and exact *P* values.

Cytokines and chemokines play important roles in responding to infection, in most pwCF and in model infection systems, yet the inflammatory responses to chronic *P. aeruginosa* infection in *Scnn1b-*Tg mice are largely unknown. We hypothesized that *P. aeruginosa* infections would lead to increases in pro-inflammatory cytokines and neutrophil-attracting chemokines, as has been shown in some pwCF (46). We performed a 29-plex cytokine array on whole mouse lungs from infected SCFM2-*Scnn1b*-Tg and SCFM2-C57BL/6 mice and sterile agar bead controls to quantify inflammatory signals. As expected, pro-inflammatory cytokines like IL-6, IL-1β, and TNFα were upregulated during infection, while TNFα was higher in SCFM2-*Scnn1b*-Tg infected mice than SCFM2-C57BL/6 infected mice (Fig. 5A and B). The monocyte/macrophage chemokines MIP-1α and CXCL10, as well as the neutrophil chemokines KC/GRO and MIP-2, were significantly upregulated in infected SCFM2-*Scnn1b*-Tg mice relative to sterile agar bead controls (Fig. 5A, C and D), with MIP-1α, KC/GRO, and MIP-2 higher in SCFM2-*Scnn1b*-Tg infected mice than SCFM2-C57BL/6 infected mice (Fig. 5C and D). Furthermore, there was a significant interaction between the infection with *P. aeruginosa* and the *Scnn1b*-Tg genotype on KC/GRO levels (Table S1), demonstrating a synergistic effect of these parameters on the neutrophil-attractant chemokine. The increased inflammatory cytokines and chemokines in SCMF2-*Scnn1b*-Tg mice are surprising since the counts for most of the cell types of the innate response were not different between infected SCFM2-*Scnn1b*-Tg and WT SCFM2-C57BL/6 mice (Fig. 3). This could mean that although the cell numbers are similar, innate cells in SCFM2-*Scnn1b*-Tg mice are hyperactivated by infection, leading to increased cytokine production.

Given the differences in T cell populations during the SCFM2-*Scnn1b-*Tg and SCFM2-C57BL/6 infections, we were interested in how T cell cytokines were modulated during chronic infection (Fig. 5A and E). MIP-3α, a chemokine expressed by activated macrophages and a strong chemoattractant for lymphocytes (47), was increased during infections of both mice genotypes relative to sterile agar beads and was significantly higher in SCFM2-*Scnn1b*-Tg than SCFM2-C57BL/6 infected mice. IL-15, which promotes CD8^+^ T cell proliferation (48), was only increased in WT mice, while IL-16, a major CD4^+^ T cell activator (49), was significantly increased only in infected SCFM2-*Scnn1b*-Tg mice relative to sterile agar bead controls (Fig. 5E). The increase of MIP-3α and IL-16 may explain the higher numbers of effector T cells seen in infected SCFM2-*Scnn1b*-Tg mice compared to infected WT mice (Fig. 4).

### Chronic infection leads to altered lymphoid-mediated inflammation in *Scnn1b*-Tg mice

To further understand the type of inflammation during *P. aeruginosa* chronic infection in our model, we measured levels of typical cytokines present during different types of inflammation, including Th1, Th2, and Th17.

**Fig 6:**
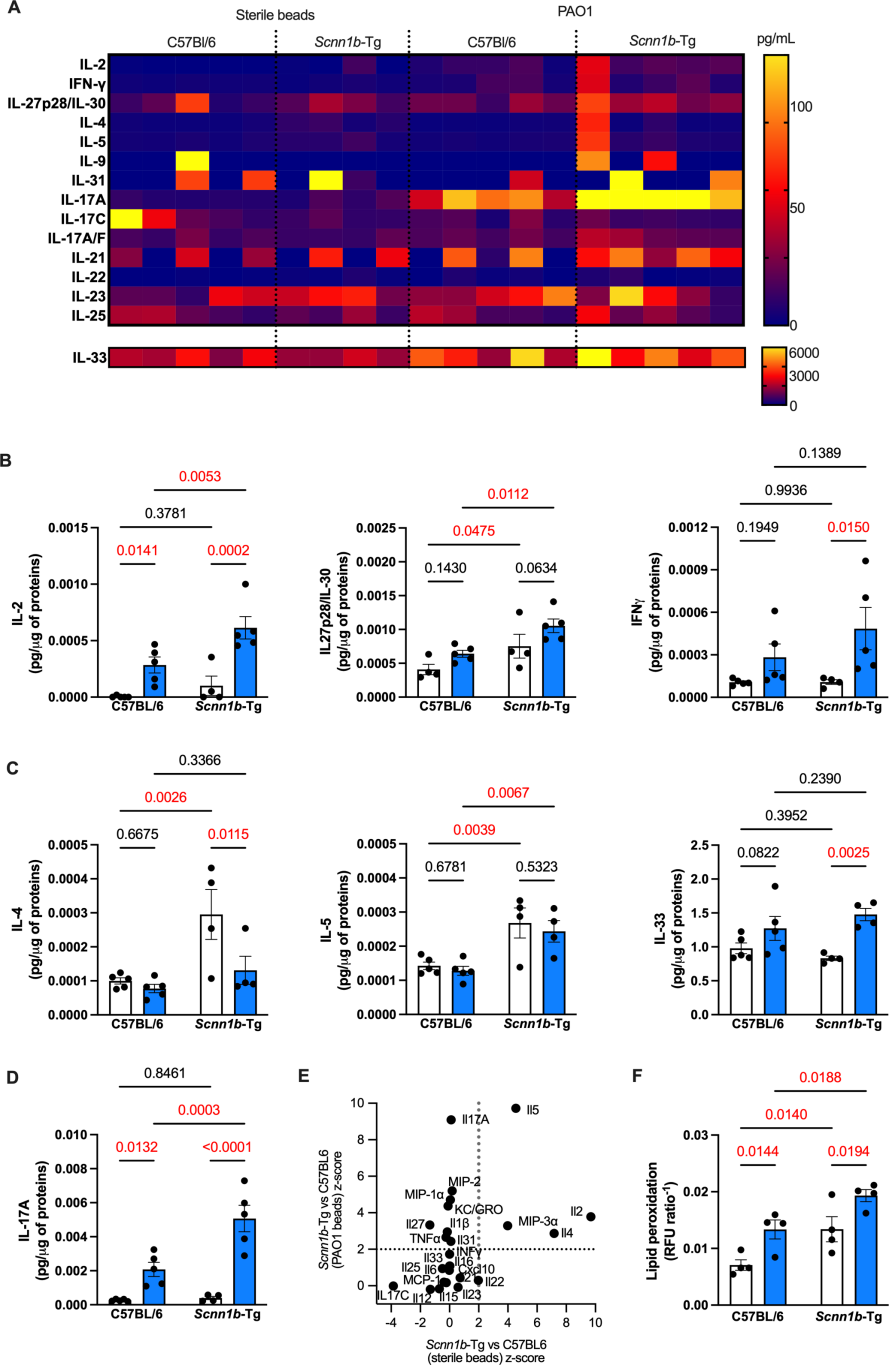
Chronic infection leads to dysfunctional lymphoid-mediated inflammation in SCFM2-*Scnn1b*-Tg mice. (**A**) Quantification (pg/μg of proteins) of types 1, 2, and 3 inflammation cytokines and chemokines in whole lung lysates of chronically infected mice. (**B**) Type 1 inflammation lymphokines IL-2, IL-27p/28/IL-30, and IFN-γ were significantly upregulated in infected *Scnn1b*-Tg mice. (**C**) Type 2 inflammation lymphokines IL-4 and IL-5 were upregulated in uninfected *Scnn1b*-Tg mice. Although IL-4 was downregulated during infection, IL-5 levels were maintained. IL-33 was upregulated in *Scnn1b*-Tg mice during chronic infection. (**D**) Type 3 inflammation cytokine IL-17 was upregulated in all mice and further increased in *Scnn1b*-Tg mice. (**E**) *Z*-scores highlight type 2 and lymphoid inflammation in uninfected *Scnn1b*-Tg mice compared to their WT littermates. IL-17 is the most differentially upregulated cytokine in these mice during infection. (**F**) *Scnn1b*-Tg have higher lung tissue damage at baseline. Chronic infection caused increased lipid peroxidation in all infected mice but was greater in *Scnn1b*-Tg mice. For (B–D and F), white bars indicate sterile SCFM2 agar bead controls, blue bars indicate SCFM2 agar beads with PAO1, *n* = 4–5 mice/group, *P < 0.05, **P < 0.01, ***P < 0.001, ****P < 0.0001. See Table S1 for statistical tests used and exact *P* values.

Type 1 inflammation is driven by Th1 lymphocytes and triggered in response to harmful pathogens or injury (50). IL-2, which promotes the survival and differentiation of naïve T cells in Th1 and Th2 (51, 52), was upregulated during infection and increased in infected SCFM2-*Scnn1b*-Tg mice relative to WT mice (Fig. 6A and B). IFNγ and IL-27 are secreted during this type 1 response. IL-27 was upregulated in infected SCMF2-*Scnn1b*-Tg mice relative to WT, while IFNγ was upregulated in infected SCMF2-*Scnn1b*-Tg mice relative to sterile agar bead controls (Fig. 6A and B). Type 1 inflammatory response to bacterial infection is expected, but these results support an exacerbated response to infection in SCFM2-*Scnn1b*-Tg mice. We also measured cytokines involved in types 2 and 3 inflammation. Type 2 inflammation is an overactive immune response common in asthmatic and allergic diseases (53), is correlated with decreased lung function, and is common during *P. aeruginosa* infections in CF (54).

Type 2 inflammation is characterized by eosinophilia and high levels of IL-4, IL-5, and IL-33 (50, 55). Our cytokine array revealed that IL-4 and IL-5 levels were significantly higher in uninfected *Scnn1b*-Tg mice compared to WT mice (Fig. 6A and C). This was consistent with the presence of eosinophils in the BAL of uninfected *Scnn1b*-Tg mice (Fig. S2). IL-4 is secreted by Th2 lymphocytes, eosinophils, basophils, and mast cells and induces differentiation of naïve helper cells into Th2 lymphocytes (56). IL-5 is produced by Th2 cells and is a key mediator of eosinophil activation (57). These results support the presence of chronic type 2 inflammation in *Scnn1b*-Tg mice even before infection. During chronic infection, it was surprising to see that while IL-4 decreased, IL-5 levels did not change (Fig. 6A and C). IL-33, another cytokine involved in the maturation of Th2 cells and activation of eosinophils (58), was increased during the SCMF2-*Scnn1b*-Tg chronic infection specifically (Fig. 6A and C).

IL-17A is a cytokine produced by Th17 cells and plays a key role in T cell-mediated neutrophil mobilization and activation (50, 59, 60). IL-17-mediated inflammation has been correlated with pulmonary exacerbations and *P. aeruginosa* infections in pwCF (61, 62). IL-17A was upregulated in all infected mice but was significantly higher in the lungs of infected SCFM2-*Scnn1b*-Tg mice compared to WT SCFM2-C57BL/6 mice (Fig. 6A and D). Furthermore, as for KC/GRO, there was a significant interaction between the infection with *P. aeruginosa* and the *Scnn1b*-Tg genotype on IL-17A levels, demonstrating a synergistic effect of these parameters on the neutrophil-attractant chemokine (Table S1).

Finally, we used *z*-scores to comprehensively compare *Scnn1b*-Tg and WT C57BL/6 inflammation in infected mice and uninfected sterile agar beads controls (Fig. 6E). The high *z*-scores of IL-2, MIP-3α, IL-4, and IL-5 further highlight the activation of T cell-mediated type 2 inflammation in uninfected *Scnn1b*-Tg mice, while type 3 inflammation (IL-17A) was most differentially upregulated during chronic infection in the SCMF2-*Scnn1b*-Tg mice (Fig. 6E). These results underscore the key interplay between activated T cells, eosinophils, and neutrophils during chronic *P. aeruginosa* infection and demonstrate a complex inflammatory response in our animal model that recapitulates human CF disease.

### Infection of SCFM2-*Scnn1b*-Tg mice leads to increased lung cell damage

Exacerbated chronic inflammation is known to induce high oxidative stress resulting in tissue damage in lung diseases (63). Lipid peroxidation is a measure of cellular damage and is increased in the lungs of some pwCF (64–66). To test whether the high inflammatory environment induced lung tissue damage in SCFM2-*Scnn1b*-Tg mice, we measured lipid peroxidation in the whole lung tissue. Consistent with the cytokine assay, we found increased lipid peroxidation in uninfected lungs of *Scnn1b*-Tg mice relative to WT C57BL/6 mice (Fig. 6F). Chronic infection increased lipid peroxidation in both genotypes but was significantly higher after infections in SCFM2-*Scnn1b*-Tg compared to WT SCFM2-C57BL/6 mice (Fig. 6F). These results confirmed increased tissue damage at baseline and during chronic infection in our mouse model, similar to what is found in human CF disease.

## DISCUSSION

Establishing a murine model of *P. aeruginosa* chronic infection that mimics the complex CF lung environment has been challenging investigators for decades (11). Researchers have used different engineered murine models, but most of them failed to develop spontaneous chronic infections or were limited in recapitulating key aspects of CF lung pathology, especially mucus (11). In this study, we used the *Scnn1b*-Tg mouse (34, 35), a model already described to develop mucus plugs, inflammation, and obstructive disease, to mimic the CF lung environment. We also used sputum-mimicking SCFM2 medium to embed *P. aeruginosa* to further simulate the nutritional and biofilm-promoting environment found in CF airways (7). We assessed pulmonary function and the immune response in this SCFM2*-Scnn1b*-Tg model, including immune cell populations and cytokine and chemokine signaling molecules. We showed that chronic *P. aeruginosa* infection decreased inspiratory capacity and compliance, elevated airway resistance, and significantly reduced FVC and FEV0.1, which are equivalent to human FEV1 measured in clinical spirometry (67). We also demonstrated a greater susceptibility to lung function decline for SCFM2-*Scnn1b*-Tg mice compared to WT SCFM2-C57BL/6 littermates. Like human CF disease, *P. aeruginosa* infected SCFM2-*Scnn1b*-Tg mice mainly develop obstructive lung disease that is mixed with restrictive disorder, making this model ideal to test the effectiveness of therapies designed to improve lung function in pwCF.

**Fig 7:**
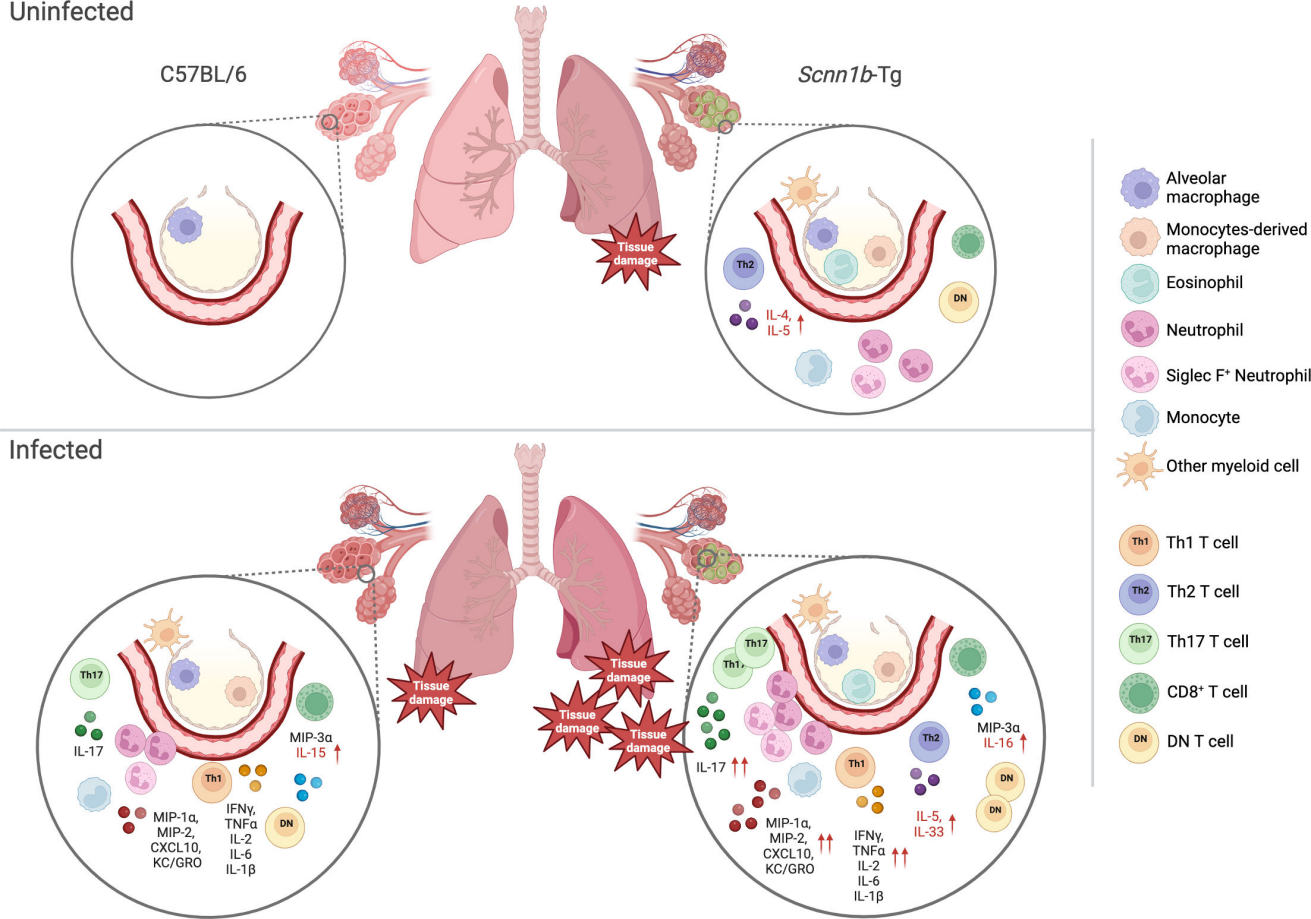
Summary of lung inflammation in SCFM2-C57BL/6 and SCFM2-*Scnn1b*-Tg mice during chronic infection with *P. aeruginosa*. Healthy lungs from C57BL/6 mice have surveilling alveolar macrophages. Uninfected *Scnn1b*-Tg mice show underlying inflammation characterized by the presence of alveolar and monocyte-derived macrophages, monocytes, other myeloid cells, and effector T cells. Conventional and Siglec F^+^ neutrophils are also present in the BAL of uninfected *Scnn1b*-Tg mice. Type 2 inflammation, demonstrated by the presence of eosinophils and IL-4 and IL-5, is present at baseline in the *Scnn1b*-Tg lung environment. During chronic infection, both SCFM2-C57BL/6 and SCFM2-*Scnn1b-*Tg immune responses are characterized by infiltration of innate and T cells and high levels of types 1 and 3 inflammation cytokines and chemokines (IL-1β, IL-2, IL-6, IL-17, TNFα, IFNγ, MIP-1α, MIP-2, MIP-3α, and KC/GRO). In addition to being exacerbated, SCFM2-*Scnn1b*-Tg inflammation is characterized by a sustained type 2 inflammation, a marked IL-17/neutrophil interplay, and the recruitment of unconventional Siglec F^+^ neutrophils. Higher inflammation is associated with higher lung tissue damage in SCFM2-*Scnn1b*-Tg mice. Cytokines in red font are cytokines expressed in the specific genotypes. Red arrows indicate where the cytokine production is increased relative to the other genotype.

In most pwCF, inflammation is a complex mix of innate and lymphoid inflammation (24). It is characterized by high secretion of pro-inflammatory cytokines like IL-6, IL-1β, and TNFα, but also type 2 (IL-4, IL-5, and IL-33) and type 3 (IL-17) inflammatory cytokines (24, 61, 68). Our characterization of the lung immune response also showed a complex inflammatory environment resembling pwCF (Fig. 7). At baseline, *Scnn1b*-Tg mice demonstrated chronic inflammation characterized by higher counts of myeloid- and lymphoid-derived cell counts in the alveolar space. During chronic infection, although the innate cell counts were similar in SCFM2-*Scnn1b*-Tg mice and their WT SCFM2-C57BL/6 mice littermates, we showed that SCFM2-*Scnn1b*-Tg mice had exacerbated inflammation which included pro-inflammatory cytokines IL-6, IL-1β, and TNFα, and chemokines MIP-1α, CXCL10, MIP-2, and KC/GRO. This suggests that monocytes, macrophages, and neutrophils may be hyperactivated in the infected SCFM2-*Scnn1b*-Tg mouse lungs. We also describe for the first time the presence of an atypical neutrophil subset positive for the surface lectin Siglec F. Little is known about these Siglec F^+^ neutrophils, but they were recently described as long life-span and high ROS activity neutrophils and were shown to be deleterious in tissue fibrosis and tumor tolerance (69–72). In a mouse nasal mucosae infection model, high IL-17 secreting Siglec F^+^ neutrophils were associated with better clearance of *Bordetella pertussis* (73). In our study, it is not clear whether the recruitment of Siglec F^+^ neutrophils is deleterious or a response to the infection to tentatively clear *P. aeruginosa*. Shin et al. also described the induction of these unique neutrophils in air pollutant-induced lung damage (74). Siglec F^+^ neutrophils were associated with exacerbated asthma and triggered emphysema by producing high levels of cysteinyl leukotrienes and neutrophil extracellular traps. *In vivo*, Siglec F^+^ neutrophils enhanced IL-5 and IL-13 production by Th2 cells and IL-17 secretion by CD4^+^ T cells (74). Here, since Siglec F^+^ neutrophils were already present in the BAL of uninfected *Scnn1b*-Tg mice and recruited during chronic infection, we hypothesize that this unique population could be involved in higher types 2 and 3 inflammatory cytokines seen in the *Scnn1b*-Tg mice during chronic infections. Presently, Siglec F^+^ neutrophils have not been described in pwCF, and it is possible that these neutrophils may represent a distinct population found only in mice and not in pwCF. However, a unique neutrophil subset called low-density neutrophils has been found in the blood of some pwCF and in other inflammatory diseases (75, 76). Like Siglec F^+^ neutrophils, low-density neutrophils have increased IL-17 production, enhanced degranulation, and decreased phagocytosis (75, 77, 78). Furthermore, low-density neutrophils were associated with pulmonary exacerbations, decreased lung function, and disease progression in some pwCF (76, 79). Although we cannot directly compare low-density neutrophils with the Siglec F^+^ neutrophils detected here, we speculate that these two neutrophil subsets may have similar effects on chronic inflammation. Since Siglec F^+^ neutrophils, but not total neutrophil counts, were increased in *Scnn1b*-Tg mice and by the infection, we speculate that this specific neutrophil subset may modulate inflammation during chronic infection with *P. aeruginosa*. To our knowledge, this is the first time these Siglec F^+^ neutrophils have been observed in a CF-like model.

Another feature of our model is the evident infiltration of effector CD4^+^ T cells at baseline and their proliferation during chronic infection in the *Scnn1b*-Tg mice. Lymphocytosis was also described in some pwCF (24, 80), and a skewed response toward Th2 and Th17 inflammation in these patients was associated with *P. aeruginosa* infection, decreased lung function, and higher mortality (54, 61). Mueller et al. demonstrated that this skewed T cell response could result from the CFTR deficiency in lymphocytes (81). Interestingly, *Scnn1b*-Tg mice, which do not lack the CFTR channel, also show Th2 and Th17 responses during chronic infection. *P. aeruginosa* toxins can also increase type 2 inflammation resulting in higher eosinophil infiltration, cytokine (IL-4 and IL-13) and IgE secretion, and mucus production (82). It is unclear whether *P. aeruginosa* had a role in the maintenance of the Th2 response in the infected SCFM2-*Scnn1b*-Tg mice since no Th2 response was induced in WT SCFM2-C57BL/6 mice (Fig. 6). This difference could suggest that either the *P. aeruginosa* secretome is modulated by the CF-like environment, or that other independent factors are responsible for the Th2 maintenance in the *Scnn1b*-Tg mice. Fritzsching et al. described spontaneous type 2 inflammation in juvenile *Scnn1b*-Tg mice caused by impaired mucus clearance (36). These mice developed an exacerbated eosinophilic response to allergen challenge. In our study, our *Scnn1b*-Tg mice also had higher baseline levels of IL-4 and IL-5 and eosinophil counts. We believe this underlying asthmatic inflammation could play a role in the exacerbated inflammatory response seen in SCFM2-*Scnn1b*-Tg mice during chronic infection. Finally, IL-17 is a key player in Th17-mediated (type 3) inflammation and T cell-mediated neutrophil mobilization and activation (50, 59, 60). IL-17-mediated inflammation was also described in some pwCF and was correlated with pulmonary exacerbations and infection with *P. aeruginosa* (61, 62*)*. The fact that KC/GRO and IL-17 were both upregulated during chronic infection in SCFM2-*Scnn1b*-Tg mice underscores the importance of the Th17/neutrophil interplay in the immune response of this model.

To resolve inflammation, regulatory T cells play an important role by secreting the anti-inflammatory cytokine IL-10. Regulatory T cells were shown to be decreased in pwCF and positively correlated with FEV1 (83). Regulatory T cells were increased in both mouse genotypes during infection, which is opposite to what has been reported for pwCF. Nevertheless, their low proportion (1/10^4^ to 1/10^3^ T cells) and the almost undetectable levels of IL-10 suggest a limited role for the regulatory T cells subset in the SCFM2-*Scnn1b-*Tg model.

Activated and central memory CD8^+^ T cells are important in type 1 inflammation in response to intracellular pathogens and were upregulated in both WT SCFM2-C57BL/6 and SCFM2-*Scnn1b*-Tg mice during chronic infection. The marked upregulation in central memory CD8^+^ T cells and IL-15 secretion in WT mice following infection suggests a role for these cells in *P. aeruginosa* clearance. In SCFM2-*Scnn1b*-Tg mice, naïve CD8^+^ T cells and CD8-derived DN T cells were significantly increased relative to WT SCFM2-C57BL/6 infected mice. Little is known about DN T cells during infection. Intracellular pathogens such as *Leishmania major* and *Francisella tularensis* have been shown to induce DN T cells in murine models and produce IFNγ, TNFα, IL-17, and granzyme B (84–86). Although these DN T cells were protective against intracellular pathogens, it is unclear whether they had a role in *P. aeruginosa* clearance in our study. To our knowledge, elevated CD8^+^ and DN T cells are not described in pwCF. Their presence during chronic infection in our mice may represent a limitation of using this model to study the inflammatory response to *P. aeruginosa* infection.

With its lung obstructive disease, underlying complex inflammation, tissue damage, and inability to clear bacterial infection, we believe our SCFM2-*Snn1b-*Tg mouse model is a suitable model to study host-pathogen interactions during chronic lung infections with *P. aeruginosa* and possibly other pathogens. One primary limitation of our model is that it does not involve a CFTR deficiency, and thus cannot be used for modeling CFTR modulator therapies or for studying how loss of CFTR may affect bacterial interactions with host cells (34, 87). Because bacterial infections tend to persist in most pwCF, even after CFTR modulator treatment (88, 89), it is important to consider alternative models for modulator-related studies, but also consider their limitations. Other rodent and non-rodent models have been used to reproduce the CF lung pathology (11, 90). CFTR-defective pigs and ferrets have similar lung pathologies to CF patients and are the only pre-clinical models to develop spontaneous lung infections (91–96). However, their severe intestinal disease, substantial cost, and the strict regulations can make these models challenging to use in research (97). Rat models, including the CFTR knockout, the F508del CFTR, and the humanized G551D models, also develop defective ion transport, airway mucus plugs, and multiorgan defects (98–101), making them appealing to study CFTR dysfunction using modulators. However, like mice, rats do not develop spontaneous lung infection, and agar bead strategies were also used in this model to establish chronic infection (102). Furthermore, although these rats showed a higher neutrophilic response to infection (101, 102), it is not known whether they develop the complex asthmatic inflammation and lymphocytosis seen in some pwCF and in the SCFM2-*Scnn1b-*Tg mouse model described here. Since our SCFM2-*Scnn1b-*Tg mouse model utilizes SCFM2, and SCFM2 was shown to induce *P. aeruginosa* transcriptional profiles similar to those in human CF sputum in normal mice (37), we are confident that this SCFM2-*Scnn1b-*Tg mouse model will be a valuable tool for investigating potential antimicrobials and the evolution of microbes during chronic infection.

## MATERIALS AND METHODS

### Study design

The objective of this study was to establish a chronic murine lung infection model in *Sccn1b-*Tg mice using SCFM2 agar beads laden with *P. aeruginosa* PAO1 and to determine the effects of this chronic infection on pulmonary function and inflammation. To determine these phenotypes, *P. aeruginosa* infected *Scnn1b-*Tg mice were compared to *P. aeruginosa* infected WT C57BL/6 mouse littermates, and sterile SCFM2 agar beads were also used as controls in both mouse genotypes to account for any possible bead-specific effects. In addition, baseline analyses were performed on untreated *Scnn1b-*Tg mice and WT C57BL/6 mouse littermates. For all mouse experiments, four to six mice were used per group. Pulmonary function in each treatment group was measured as described below. Inflammation was determined by measuring immune cell populations by flow cytometry analyses, cytokine production, and lipid peroxidation. Mice were randomly assigned to groups, including equal distributions of males and females.

### Mouse model

B6N.Cg-Tg(Scgb1a1-Scnn1b)6608Bouc/J mice (103), herein named *Scnn1b-*Tg, were purchased from Jackson Laboratories (JAX stock #030949) and bred with C57BL/6 mice. Males and females 8–12 weeks old were equally distributed between the groups for experiments. *Scnn1b-*Tg mice were compared with their WT littermates. A total of 31 *Scnn1b-*Tg and 29 WT mice were used for experiments, divided into four to six mice/group. The Cedars-Sinai Institutional Animal Care and Use Committee approved all experiments according to current NIH guidelines.

### *P. aeruginosa* embedding in SCFM2-agar beads

*P. aeruginosa* strain PAO1 (parental strain for the Manoil lab two-allele transposon mutant library [40]) was obtained from Pradeep K. Singh (30) and grown in SCFM2 medium (7, 8). PAO1 embedding in SCFM2-agar beads was performed using a protocol adapted from Facchini et al. (104). Briefly, a single colony was inoculated into 3 mL SCFM2 and incubated at 37°C overnight in a shaking incubator at 250 rpm. The next day, the culture was diluted in 7 mL of fresh SCFM2 and grown until a total of ~5 OD was reached. In the meantime, 3% Bacto agar and 50 mL of heavy mineral oil were autoclaved at 121°C for 45 min and equilibrated at 50°C in a water bath. Bacto agar was then mixed with 2× SCFM2 pre-equilibrated at 50°C in a 1:1 ratio. Bacterial suspension was spun down, resuspended in 300 µL sterile PBS, and mixed with 3 mL of 1.5% Bacto agar 1× SCFM2 solution. The SCFM2 agar-*P. aeruginosa* mixture was added to heavy mineral oil and immediately stirred for 6 min at room temperature. The mixture was cooled to 4°C by stirring in iced water for 30 min. Agar beads were then transferred into 50 mL Falcon tubes and centrifuged at max speed for 15 min at 4°C. Mineral oil was removed, and agar beads were washed with sterile PBS four times. After the last wash, agar beads were resuspended in 25 mL PBS. To calculate the bacterial load of agar beads, an aliquot of the beads (approximately 0.5 mL) was aseptically homogenized and serially diluted 1:10 down to 10^−6^. Each dilution was spotted on LB plates and incubated at 37°C overnight. The beads were stored at 4°C until the infection.

### Chronic infection

*Scnn1b-*Tg mice and WT littermates were anesthetized using 4% isoflurane. Sterile agar beads or 1 × 10^6^ CFU *P*. *aeruginosa*-laden SCFM2-agar beads were inoculated intratracheally using a 22-gauge angiocatheter (*n* = 4–6 mice/ group). After 7 days of infection, in experiment 1, lung function measurements, cytokine analyses, and CFU count were performed, and in experiment 2 flow cytometry was performed.

### Lung function measurements

The lung function was assessed by forced oscillation techniques (FOTs) and forced expiratory using the flexiVent FX system (SCIREQ) (105). The system was equipped with an FX2 module as well as with a NPFE extension for mice, and it was operated by the flexiWare v7.2 software. Mice were anesthetized with isoflurane, intubated with an 18- to 20-gauge angiocatheter, and placed in the supine position in a plethysmograph chamber. Mice were mechanically ventilated at a tidal volume of 10 mL/kg and frequency of 150 breath/min. The perturbations performed were deep inflation, FOT, PV loop, and NPFE.

### CFU counts

After euthanasia, lungs were harvested and homogenized in sterile PBS using the Bead Mill 24 Homogenizer (Fisherbrand). The mixture was serially diluted 1:10 down to 10^−6^. Each dilution was spotted on LB plates and incubated at 37°C overnight. CFUs were then counted and reported as CFUs/mL.

### BAL for flow cytometry

BAL was performed with 6 × 1 mL sterile cold 2 mM EDTA/2% FBS/1× PBS buffer. BAL was spun at 500 rcf for 10 min at 4°C. Cell pellets were then resuspended in 3 mL RBC Lysis buffer and incubated at room temperature for 3 min. RBC lysis was stopped by adding 30 mL cold 3% FBS/1× PBS buffer. Cells were spun down, resuspended in 1.5 mL cold 3% FBS/1× PBS buffer, and counted using the TC20 Automated Cell Counter (Bio-Rad).

### Lung digestion for flow cytometry

Lungs were perfused through the right ventricle with 10 mL 1× PBS to flush blood out of lung tissue. Lungs were then removed, minced, and digested in 11 mL 0.2% collagenase II (Worthington Biochem cat# LS004176)/10% FBS/RPMI 1640 media in a 37°C incubator shaking at 250 rpm for 30 min. Digested lungs were then strained through a 70 µm cell strainer and spun down at 500 rcf for 10 min at 4°C. Cell pellets were then resuspended in 3 mL RBC Lysis buffer and incubated at room temperature for 3 min. RBC lysis was stopped by adding 30 mL cold 3% FBS/1× PBS buffer. Cells were spun down, resuspended in 5 mL cold 3% FBS/1× PBS buffer, and counted using the TC20 Automated Cell Counter (Bio-Rad). Cells were separated into different aliquots for inflammatory panel and lipid peroxidation assay.

### Inflammatory panel by flow cytometry

Up to 5 × 10^6^ cells were spun down in 1.5 mL tubes at 8,000 rcf for 1 min. Pellets were resuspended in 50 µL 3% FBS/1× PBS buffer with 2 µL FC block (BD cat# 553141) and incubated on ice for 20 min. Next, 50 µL of 3% FBS/1× PBS containing 0.25 µL of each cell surface antibody (see Table S2) was added, and tubes were incubated on ice for 30 min in the dark. Cells were washed with 1 mL 3% FBS/1× PBS buffer and spun down. Cell pellets were then fixed in 500 µL cold 2% PFA and incubated at room temperature for 10 min with occasional vortexing to maintain single-cell suspension. Cells were spun down and washed with 1 mL 3% FBS/1× PBS buffer. Cells were permeabilized in 150 µL 0.2% Tween-20/1× PBS buffer and incubated at room temperature for 15 min in the dark. Then, 50 µL of 0.2% Tween-20/1× PBS containing 1 µL of PE-Foxp3 (Miltenyi Biotec cat# 130-111-678) was added, and cells were incubated for 30 min in the dark. Cells were washed with 1 mL 3% FBS/1× PBS buffer, spun down, and resuspended in 400 µL 1X PBS. Cell suspensions were filtered through a 70 µm mesh before analyzing on the Cytek Aurora spectral flow cytometer. Unmixing was performed with the Cytek SpectroFlo software version 3.1.0, and cell populations were analyzed on BD FlowJo version 10.8.2 and determined as follows (Fig. 3): inflammatory cells (CD45^+^), neutrophils (Ly6G^+^), eosinophils (CD11b^+^, CD11c^−^, and Siglec-F^+^), alveolar macrophages (Siglec-F^+^ and CD11c^+^), classical monocytes (CD11b^+^ and Ly6C^+^), monocyte-derived macrophages (CD11b^High^, Ly6C^+/−^, CD64^+^, and FSC-A^high^), other myeloid-derived cells (CD11b^+^ and Ly6C^−^), T cells (TCRβ^+^), T helper (TCRβ^+^ and CD4^+^), Treg (TCRβ^+^, CD4^+^, and Foxp3^+^), cytotoxic T cells (TCRβ^+^ and CD8^+^), DN T cells (TCRβ^+^, CD4^−^, and CD8^−^), naïve T cells (TCRβ^+^, CD44^−^, and CD62L^+^), effector T cells (TCRβ^+^, CD44^+^, and CD62L^−^), central memory T cells (TCRβ^+^, CD44^+^, and CD62L^+^). The inflammatory antibody panel (Table S2) was designed using the EasyPanel V2 software (Omiq, LLC).

### Cytokine array

Total proteins were extracted from frozen lung tissue using Meso Scale Discovery MSD Tris Lysis Buffer (MSD cat# R60TX-3) supplemented with Protease Inhibitor Cocktail (Thermo Scientific cat# 78425), Phosphate Inhibitor Cocktail 2 (Sigma cat# P5726) and Phosphate Inhibitor Cocktail 2 (Sigma cat# P0044). Proteins were quantified using the BCA Protein Assay (Genesee Scientific cat# 18-440). Cytokine array was performed using the V-PLEX Mouse Cytokine 29-Plex Kit (Meso Scale Discovery cat# K15267D-1). The following cytokines and chemokines were included: IFN-γ, IL-1β, IL-2, IL-4, IL-5, IL-6, IL-9, IL-10, IL-12p70, IL-15, IL-16, IL-17A, IL-17A/F, IL-17C, IL-17E/IL-25, IL-17F, IL-21, IL-22, IL-23, IL-27p28/IL-30, IL-31, IL-33, CXCL10, KC/GRO, MCP-1, MIP-1α, MIP-2, MIP-3α, and TNF-α. The assay was performed following the manufacturer’s instructions and was analyzed on the Meso Scale Discovery instrument. Cytokine and chemokine levels were normalized to total proteins for the statistical analyses.

### Lipid peroxidation assay

Up to 5 × 10^6^ cells were spun down in 1.5 mL tubes at 8,000 rcf for 1 min. Pellets were resuspended in 100 µL of lipid peroxidation reagent 1:500 (Abcam cat# ab243377) and incubated for 30 min at 37°C and 5% CO_2_. Cells were washed with 1 mL 3% FBS/1× PBS buffer, spun down, and resuspended in 400 µL 1× PBS. Cell suspensions were filtered through a 70 µm mesh before analyzing on the BD Fortessa flow cytometer. Mean fluorescence intensity was quantified using FlowJo software. Lipid peroxidation was quantified by calculating the red (Ex561/Em582)/green (Ex488/Em525) fluorescence ratio. Data are presented as the reciprocal of the ratio (1/ratio).

### Statistical analysis

Normality and homogeneity of variance were assessed by Shapiro-Wilk and Brown-Forsythe tests, respectively. Data were log-transformed prior to analysis where necessary to meet assumptions necessary for parametric testing; otherwise, non-parametric rank testing was used. Based on data distributions, analyses between two groups were performed using Student’s *t*-test or the nonparametric Mann-Whitney test. To detect any possible interaction between the mouse genotype and the infection on the parameters, ordinary two-way ANOVA followed by Tukey’s post hoc test was used for comparisons between the four groups. Significant outliers determined by the Graph Pad Outlier Calculator were removed from statistics. All testing was considered significant at the two-tailed *P* value of <0.05. Analysis performed with GraphPad Prism v10. The *P* values are listed in Table S1.

## SUPPLEMENTAL MATERIAL

Supplemental material iai.00230-25-s0001.pdf Fig S1 to S4; Tables S1 and S2.

ASM does not own the copyrights to Supplemental Material that may be linked to, or accessed through, an article. The authors have granted ASM a non-exclusive, world-wide license to publish the Supplemental Material files. Please contact the corresponding author directly for reuse.
