# Supplementary material for: A chronic Pseudomonas aeruginosa mouse lung infection modeling the mucus obstruction, lung function, and inflammation of human cystic fibrosis
Source: Infect Immun. 2025 Jun 13;93(7):e00230-25. doi: 10.1128/iai.00230-25 (PMC12234440; doi:10.1128/iai.00230-25)
Supplement: Supplemental material — Fig S1 to S4; Tables S1 and S2. [file iai.00230-25-s0001.pdf]

Supplemental Material

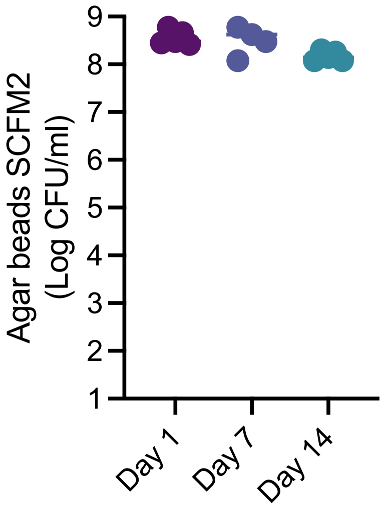

Figure S1. PAO1 remains viable in SCFM2 agar beads for at least two weeks. *P. aeruginosa* PAO1 were embedded in SCFM2 agar beads and stored at 4°C for up to 14 days. Bacterial viability was determined by homogenizing beads and performing CFU plate counts at 1, 7, and 14 days after preparation.

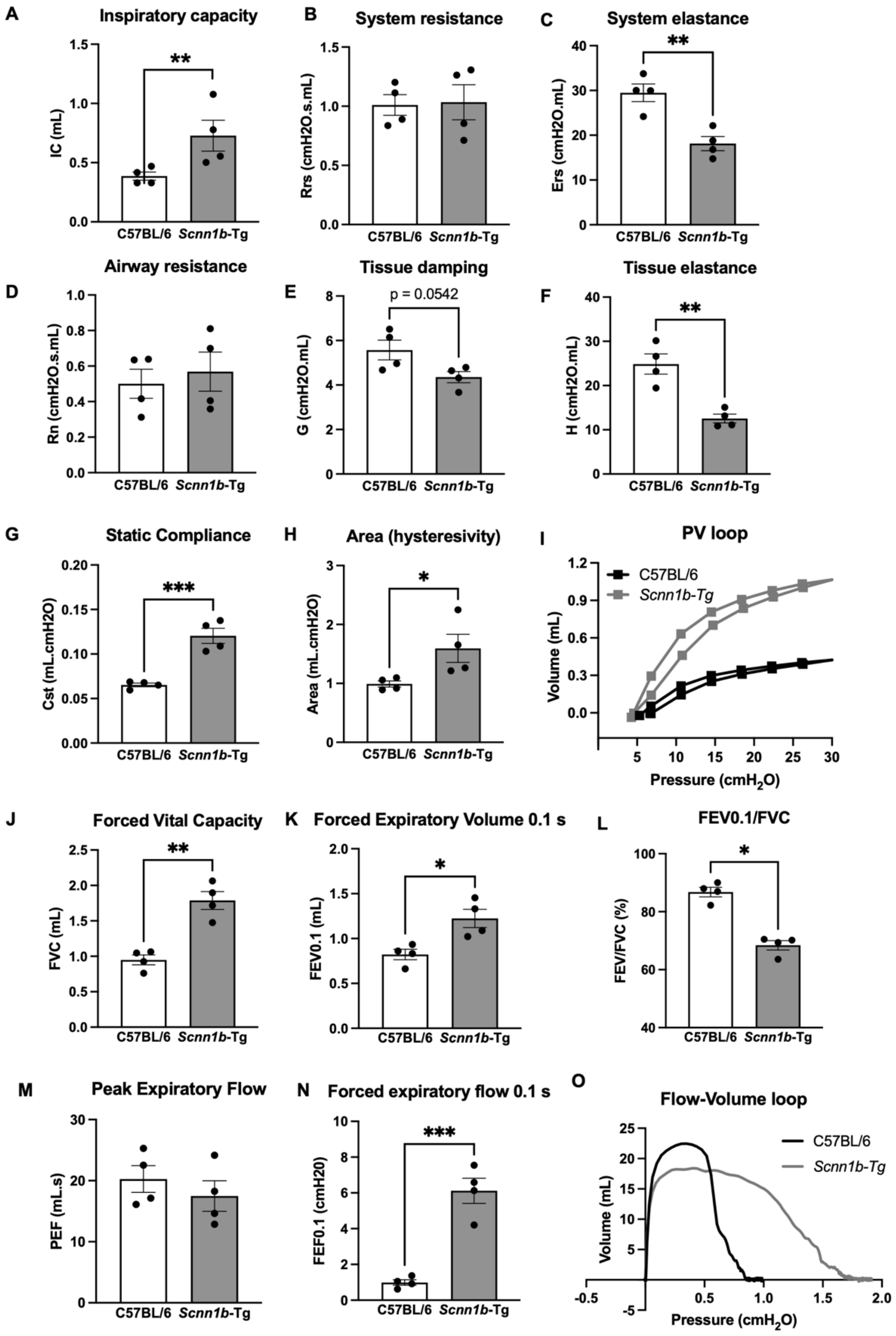

Figure S2. *Scnn1b*-Tg mice display an obstructive lung disease at baseline. Control uninfected 8-week-old WT C57BL/6 or *Scnn1b*-Tg mice were subjected to flexiVent (SCIREQ) for

baseline lung function measurements. (**A**) Inspiratory capacity using a deep inflation technique. (**B-C**) System resistance (**B**) and elastance (**C**) parameters acquired by the single frequency forced oscillation maneuver. (**D-F**) Airway resistance (**D**), tissue resistance (damping) (**E**), and elastance (**F**) obtained from the low frequency forced oscillation technique. (**G-H**) Static compliance (**G**) and hysteresivity (**H**) obtained by a pressure-volume (PV) loop. (**I**) Representative image of PV-loop. (**J-N**) FVC (**J**), FEV0.1 (**K**), FEV0.1/FVC (**L**), PEF (**M**), and FEF0.1 (**N**) obtained from the forced expiratory volume perturbation. (**O**) Representative image of the forced expiratory volume perturbation. n=4 mice/group. *$p<0.05$, **$p<0.01$, ***$p<0.001$. See Table S1 for statistical tests used and exact *p*-values.

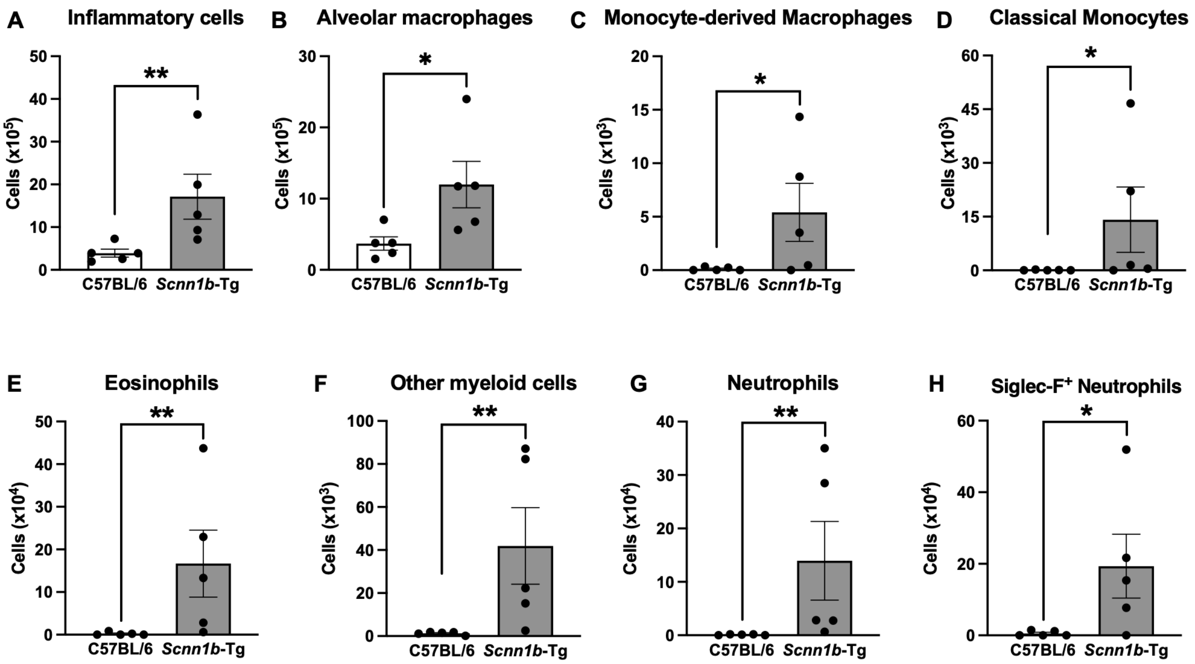

Figure S3. Uninfected *Scnn1b*-Tg mice develop an underlying lung inflammation. BAL was collected from control uninfected 8 to 12-week-old WT C57BL/6 or *Scnn1b*-Tg mice for flow cytometry. **A.** Inflammatory cells were increased in *Scnn1b*-Tg mice at baseline. **B-G.** Different innate cells were upregulated in uninfected *Scnn1b*-Tg mice. **B.** Alveolar macrophages. **C.** Monocyte-derived macrophages. **D.** Classical monocytes. **E.** Eosinophils. **F.** Other myeloid cells. **G.** Neutrophils. **H.** An atypical Siglec F^{+} neutrophil subset was present in *Scnn1b*-Tg mice at baseline. n=5 mice/group *p<0.05, **p<0.01. See Table S1 for statistical tests used and exact *p*-values.

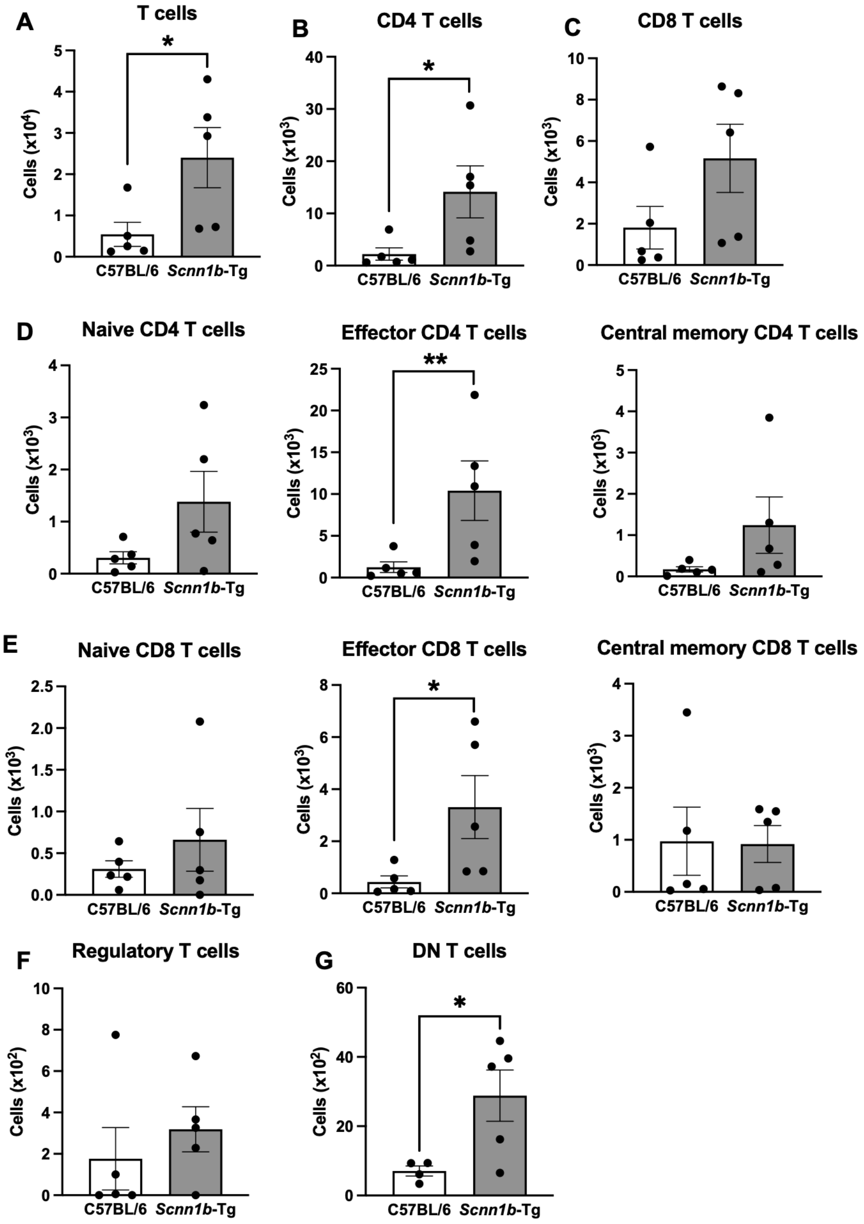

Figure S4. Lymphocytosis is present in uninfected *Scnn1b*-Tg mice. (**A**) Total T cells were significantly increased in the *Scnn1b*-Tg mice. (**B-C**) This increase in T cells was explained by higher numbers of $CD4^+$ (**B**), but not $CD8^+$ (**C**) T cells. (**D**) Activation state of $CD4^+$ T cells. A significant upregulation of effector $CD4^+$ T cells was observed in *Scnn1b*-Tg mice. Modest but

non-significant increases were also observed for naïve and central memory $CD4^+$ T cells. (**E**) Activation state of $CD8^+$ T cells. A significant upregulation of effector $CD8^+$ T cells was observed in *Scnn1b*-Tg mice. Naïve and central memory $CD8^+$ T cells were not changed compared to WT littermates. (**F**) Regulatory T cells were not increased in *Scnn1b*-Tg mice. (**G**) Double-negative (DN) cells were significantly increased in *Scnn1b*-Tg. n=5 mice/group *$p<0.05$, **$p<0.01$. See Table S1 for statistical tests used and exact *p*-values.

Table S1. Statistics.

Figure	Statistical test	Source of variation	P value	Comparison	P value
Fig 1C	Mann-Whitney	-	-	C57BL/6 PAO1 beads vs<br>Scnn1b-Tg PAO1 beads	0.0317
Fig 1D	Ordinary Two-way ANOVA	Interaction	0.1044	C57BL/6<br>Sterile vs PAO1 beads	0.9590
		Genotype	0.0031	*Scnn1b*-Tg<br>Sterile vs. PAO1 beads	0.0388
		Infection	0.1188	Sterile beads<br>C57BL/6 vs *Scnn1b*-Tg	0.0022
				PAO1 beads<br>C57BL/6 vs *Scnn1b*-Tg	0.2165
Fig 1E<br>Resistance	Ordinary Two-way ANOVA	Interaction	0.1948	C57BL/6<br>Sterile vs PAO1 beads	0.5627
		Genotype	0.5564	*Scnn1b*-Tg<br>Sterile vs. PAO1 beads	0.2157
		Infection	0.5769	Sterile beads<br>C57BL/6 vs *Scnn1b*-Tg	0.5999
				PAO1 beads<br>C57BL/6 vs *Scnn1b*-Tg	0.1865
Fig 1F<br>Elastance	Ordinary Two-way ANOVA	Interaction	0.1838	C57BL/6<br>Sterile vs PAO1 beads	0.5522
		Genotype	0.0585	*Scnn1b*-Tg<br>Sterile vs. PAO1 beads	0.2044
		Infection	0.5671	Sterile beads<br>C57BL/6 vs *Scnn1b*-Tg	0.0283
				PAO1 beads<br>C57BL/6 vs *Scnn1b*-Tg	0.6471
Fig 1G<br>Airway resistance	Ordinary Two-way ANOVA	Interaction	0.0309	C57BL/6<br>Sterile vs PAO1 beads	0.7328
		Genotype	0.2503	*Scnn1b*-Tg<br>Sterile vs. PAO1 beads	0.0115
		Infection	0.0735	Sterile beads<br>C57BL/6 vs *Scnn1b*-Tg	0.4106
				PAO1 beads<br>C57BL/6 vs *Scnn1b*-Tg	0.0234
Fig 1H<br>Tissue damping	Ordinary Two-way ANOVA	Interaction	0.3063	C57BL/6<br>Sterile vs PAO1 beads	0.6837
		Genotype	0.7906	*Scnn1b*-Tg<br>Sterile vs. PAO1 beads	0.3104
		Infection	0.6200	Sterile beads<br>C57BL/6 vs *Scnn1b*-Tg	0.5847
				PAO1 beads	0.3621

				C57BL/6 vs *Scnn1b*-Tg	
Fig 1I<br>Tissue elastance	Ordinary Two-way ANOVA	Interaction	0.1208	C57BL/6<br>Sterile vs PAO1 beads	0.5647
		Genotype	0.0282	*Scnn1b*-Tg<br>Sterile vs. PAO1 beads	0.1134
		Infection	0.4015	Sterile beads<br>C57BL/6 vs *Scnn1b*-Tg	0.0117
				PAO1 beads<br>C57BL/6 vs *Scnn1b*-Tg	0.5827
Fig 1K<br>Compliance	Ordinary Two-way ANOVA	Interaction	0.0945	C57BL/6<br>Sterile vs PAO1 beads	0.7843
		Genotype	0.0033	*Scnn1b*-Tg<br>Sterile vs. PAO1 beads	0.0489
		Infection	0.1770	Sterile beads<br>C57BL/6 vs *Scnn1b*-Tg	0.0021
				PAO1 beads<br>C57BL/6 vs *Scnn1b*-Tg	0.2399
Figure 1L<br>Hysteresis	Ordinary Two-way ANOVA	Interaction	0.0718	C57BL/6<br>Sterile vs PAO1 beads	0.6777
		Genotype	0.0022	*Scnn1b*-Tg<br>Sterile vs. PAO1 beads	0.0424
		Infection	0.1888	Sterile beads<br>C57BL/6 vs *Scnn1b*-Tg	0.0013
				PAO1 beads<br>C57BL/6 vs *Scnn1b*-Tg	0.2272
Fig 1N<br>FVC	Ordinary Two-way ANOVA	Interaction	0.0833	C57BL/6<br>Sterile vs PAO1 beads	0.7307
		Genotype	0.0047	*Scnn1b*-Tg<br>Sterile vs. PAO1 beads	0.0462
		Infection	0.1843	Sterile beads<br>C57BL/6 vs *Scnn1b*-Tg	0.0024
				PAO1 beads<br>C57BL/6 vs *Scnn1b*-Tg	0.3099
Fig 1O<br>FEV0.1	Ordinary Two-way ANOVA	Interaction	0.0388	C57BL/6<br>Sterile vs PAO1 beads	0.6316
		Genotype	0.0228	*Scnn1b*-Tg<br>Sterile vs. PAO1 beads	0.0201
		Infection	0.1261	Sterile beads<br>C57BL/6 vs *Scnn1b*-Tg	0.0041
				PAO1 beads<br>C57BL/6 vs *Scnn1b*-Tg	0.8469
Fig 1P<br>FEV0.1/FVC	Ordinary Two-way ANOVA	Interaction	0.4393	C57BL/6<br>Sterile vs PAO1 beads	0.7424
		Genotype	<0.0001	*Scnn1b*-Tg<br>Sterile vs. PAO1 beads	0.4552
		Infection	0.7323	Sterile beads<br>C57BL/6 vs *Scnn1b*-Tg	0.0056

				PAO1 beads<br>C57BL/6 vs *Scnn1b*-Tg	0.0006
Fig 1Q<br>PEF	Ordinary Two-way ANOVA	Interaction	0.0200	C57BL/6<br>Sterile vs PAO1 beads	0.2388
		Genotype	0.0447	*Scnn1b*-Tg<br>Sterile vs. PAO1 beads	0.0297
		Infection	0.3422	Sterile beads<br>C57BL/6 vs *Scnn1b*-Tg	0.7710
				PAO1 beads<br>C57BL/6 vs *Scnn1b*-Tg	0.0042
Fig 1R<br>FEF0.1	Ordinary Two-way ANOVA	Interaction	0.1307	C57BL/6<br>Sterile vs PAO1 beads	0.6993
		Genotype	<0.0001	*Scnn1b*-Tg<br>Sterile vs. PAO1 beads	0.0251
		Infection	0.0513	Sterile beads<br>C57BL/6 vs *Scnn1b*-Tg	<0.0001
				PAO1 beads<br>C57BL/6 vs *Scnn1b*-Tg	0.0002
Fig 3A	Ordinary Two-way ANOVA	Interaction	0.7406	C57BL/6<br>Sterile vs PAO1 beads	0.0024
		Genotype	0.0794	*Scnn1b*-Tg<br>Sterile vs. PAO1 beads	0.0005
		Infection	<0.0001	Sterile beads<br>C57BL/6 vs *Scnn1b*-Tg	0.2852
				PAO1 beads<br>C57BL/6 vs *Scnn1b*-Tg	0.1468
Fig 3B	Ordinary Two-way ANOVA	Interaction	0.5247	C57BL/6<br>Sterile vs PAO1 beads	0.0016
		Genotype	0.2639	*Scnn1b*-Tg<br>Sterile vs. PAO1 beads	0.0129
		Infection	0.0002	Sterile beads<br>C57BL/6 vs *Scnn1b*-Tg	0.2184
				PAO1 beads<br>C57BL/6 vs *Scnn1b*-Tg	0.7263
Fig 3C	Ordinary Two-way ANOVA	Interaction	0.2713	C57BL/6<br>Sterile vs PAO1 beads	<0.0001
		Genotype	0.3022	*Scnn1b*-Tg<br>Sterile vs. PAO1 beads	<0.0001
		Infection	<0.0001	Sterile beads<br>C57BL/6 vs *Scnn1b*-Tg	0.9596
				PAO1 beads<br>C57BL/6 vs *Scnn1b*-Tg	0.1371
Fig 3D	Ordinary Two-way ANOVA	Interaction	0.6756	C57BL/6<br>Sterile vs PAO1 beads	0.2812
		Genotype	0.0310	*Scnn1b*-Tg<br>Sterile vs. PAO1 beads	0.0912
		Infection	0.0565	Sterile beads<br>C57BL/6 vs *Scnn1b*-Tg	0.1836
				PAO1 beads<br>C57BL/6 vs *Scnn1b*-Tg	0.0723

Fig 3E	Ordinary Two-way ANOVA	Interaction	0.8029	C57BL/6<br>Sterile vs PAO1 beads	0.0461
		Genotype	0.5577	*Scnn1b*-Tg<br>Sterile vs. PAO1 beads	0.0921
		Infection	0.0122	Sterile beads<br>C57BL/6 vs *Scnn1b*-Tg	0.8107
				PAO1 beads<br>C57BL/6 vs *Scnn1b*-Tg	0.5550
Fig 3F	Ordinary Two-way ANOVA	Interaction	0.4281	C57BL/6<br>Sterile vs PAO1 beads	0.2751
		Genotype	0.2870	*Scnn1b*-Tg<br>Sterile vs. PAO1 beads	0.9829
		Infection	0.4456	Sterile beads<br>C57BL/6 vs *Scnn1b*-Tg	0.8423
				PAO1 beads<br>C57BL/6 vs *Scnn1b*-Tg	0.1935
Fig 3G	Ordinary Two-way ANOVA	Interaction	0.2607	C57BL/6<br>Sterile vs PAO1 beads	0.0006
		Genotype	0.2038	*Scnn1b*-Tg<br>Sterile vs. PAO1 beads	0.0204
		Infection	0.0002	Sterile beads<br>C57BL/6 vs *Scnn1b*-Tg	0.0824
				PAO1 beads<br>C57BL/6 vs *Scnn1b*-Tg	0.9163
Fig 3H	Ordinary Two-way ANOVA	Interaction	0.2948	C57BL/6<br>Sterile vs PAO1 beads	0.1351
		Genotype	0.0218	*Scnn1b*-Tg<br>Sterile vs. PAO1 beads	0.0047
		Infection	0.0035	Sterile beads<br>C57BL/6 vs *Scnn1b*-Tg	0.3161
				PAO1 beads<br>C57BL/6 vs *Scnn1b*-Tg	0.0231
Fig 4A	Ordinary Two-way ANOVA	Interaction	0.3639	C57BL/6<br>Sterile vs PAO1 beads	0.0472
		Genotype	0.0109	*Scnn1b*-Tg<br>Sterile vs. PAO1 beads	0.0027
		Infection	0.0008	Sterile beads<br>C57BL/6 vs *Scnn1b*-Tg	0.1997
				PAO1 beads<br>C57BL/6 vs *Scnn1b*-Tg	0.0157
Fig 4B	Ordinary Two-way ANOVA	Interaction	0.4558	C57BL/6<br>Sterile vs PAO1 beads	0.2694
		Genotype	0.0048	*Scnn1b*-Tg<br>Sterile vs. PAO1 beads	0.0388
		Infection	0.0281	Sterile beads<br>C57BL/6 vs *Scnn1b*-Tg	0.1032
				PAO1 beads<br>C57BL/6 vs *Scnn1b*-Tg	0.0115
Fig 4C	Ordinary Two-way ANOVA	Interaction	0.4415	C57BL/6<br>Sterile vs PAO1 beads	0.0914

		Genotype	0.0169	*Scnn1b*-Tg<br>Sterile vs. PAO1 beads	0.0092
		Infection	0.0036	Sterile beads<br>C57BL/6 vs *Scnn1b*-Tg	0.2124
				PAO1 beads<br>C57BL/6 vs *Scnn1b*-Tg	0.0263
Fig 4D<br>Naïve	Ordinary Two-way ANOVA	Interaction	0.9784	C57BL/6<br>Sterile vs PAO1 beads	0.8984
		Genotype	0.2135	*Scnn1b*-Tg<br>Sterile vs. PAO1 beads	0.8681
		Infection	0.8355	Sterile beads<br>C57BL/6 vs *Scnn1b*-Tg	0.3845
				PAO1 beads<br>C57BL/6 vs *Scnn1b*-Tg	0.3645
Fig 4D<br>Effector	Ordinary Two-way ANOVA	Interaction	0.1878	C57BL/6<br>Sterile vs PAO1 beads	0.0100
		Genotype	0.0199	*Scnn1b*-Tg<br>Sterile vs. PAO1 beads	0.0001
		Infection	<0.0001	Sterile beads<br>C57BL/6 vs *Scnn1b*-Tg	0.4196
				PAO1 beads<br>C57BL/6 vs *Scnn1b*-Tg	0.0123
Fig 4D<br>Central memory	Ordinary Two-way ANOVA	Interaction	0.9065	C57BL/6<br>Sterile vs PAO1 beads	0.0707
		Genotype	0.1368	*Scnn1b*-Tg<br>Sterile vs. PAO1 beads	0.1131
		Infection	0.0207	Sterile beads<br>C57BL/6 vs *Scnn1b*-Tg	0.2404
				PAO1 beads<br>C57BL/6 vs *Scnn1b*-Tg	0.3341
Fig 4E<br>Naïve	Ordinary Two-way ANOVA	Interaction	0.4019	C57BL/6<br>Sterile vs PAO1 beads	0.4695
		Genotype	0.0208	*Scnn1b*-Tg<br>Sterile vs. PAO1 beads	0.0655
		Infection	0.0721	Sterile beads<br>C57BL/6 vs *Scnn1b*-Tg	0.2561
				PAO1 beads<br>C57BL/6 vs *Scnn1b*-Tg	0.0274
Fig 4E<br>Effector	Ordinary Two-way ANOVA	Interaction	0.6727	C57BL/6<br>Sterile vs PAO1 beads	0.0026
		Genotype	0.2778	*Scnn1b*-Tg<br>Sterile vs. PAO1 beads	0.0006
		Infection	<0.0001	Sterile beads<br>C57BL/6 vs *Scnn1b*-Tg	0.6325
				PAO1 beads<br>C57BL/6 vs *Scnn1b*-Tg	0.2878
Fig 4E<br>Central memory	Ordinary Two-way ANOVA	Interaction	0.7274	C57BL/6<br>Sterile vs PAO1 beads	0.0239

		Genotype	0.1782	*Scnn1b*-Tg<br>Sterile vs. PAO1 beads	0.0661
		Infection	0.0056	Sterile beads<br>C57BL/6 vs *Scnn1b*-Tg	0.2305
				PAO1 beads<br>C57BL/6 vs *Scnn1b*-Tg	0.4698
Fig 4F	Ordinary Two-way ANOVA	Interaction	0.9272	C57BL/6<br>Sterile vs PAO1 beads	0.0154
		Genotype	0.0341	*Scnn1b*-Tg<br>Sterile vs. PAO1 beads	0.0117
		Infection	0.0011	Sterile beads<br>C57BL/6 vs *Scnn1b*-Tg	0.1371
				PAO1 beads<br>C57BL/6 vs *Scnn1b*-Tg	0.1089
Fig 4G	Ordinary Two-way ANOVA	Interaction	0.0674	C57BL/6<br>Sterile vs PAO1 beads	0.0006
		Genotype	0.0014	*Scnn1b*-Tg<br>Sterile vs. PAO1 beads	<0.0001
		Infection	<0.0001	Sterile beads<br>C57BL/6 vs *Scnn1b*-Tg	0.2278
				PAO1 beads<br>C57BL/6 vs *Scnn1b*-Tg	0.0007
Fig 5B<br>IL-6	Ordinary Two-way ANOVA	Interaction	0.4406	C57BL/6<br>Sterile vs PAO1 beads	0.0811
		Genotype	0.3895	*Scnn1b*-Tg<br>Sterile vs. PAO1 beads	0.0121
		Infection	0.0043	Sterile beads<br>C57BL/6 vs *Scnn1b*-Tg	0.9494
				PAO1 beads<br>C57BL/6 vs *Scnn1b*-Tg	0.2401
Fig 5B<br>IL-1β	Ordinary Two-way ANOVA	Interaction	0.1280	C57BL/6<br>Sterile vs PAO1 beads	0.0037
		Genotype	0.2407	*Scnn1b*-Tg<br>Sterile vs. PAO1 beads	0.0001
		Infection	<0.0001	Sterile beads<br>C57BL/6 vs *Scnn1b*-Tg	0.7854
				PAO1 beads<br>C57BL/6 vs *Scnn1b*-Tg	0.0641
Fig 5B<br>TNFα	Ordinary Two-way ANOVA	Interaction	0.0748	C57BL/6<br>Sterile vs PAO1 beads	0.1184
		Genotype	0.2203	*Scnn1b*-Tg<br>Sterile vs. PAO1 beads	0.0008
		Infection	0.0008	Sterile beads<br>C57BL/6 vs *Scnn1b*-Tg	0.6686
				PAO1 beads<br>C57BL/6 vs *Scnn1b*-Tg	0.0343
Fig 5C<br>MIP-1α	Ordinary Two-way ANOVA	Interaction	0.1273	C57BL/6<br>Sterile vs PAO1 beads	0.4647
		Genotype	0.0920	*Scnn1b*-Tg<br>Sterile vs. PAO1 beads	0.0104

		Infection	0.0184	Sterile beads<br>C57BL/6 vs *Scnn1b*-Tg	0.9000
				PAO1 beads<br>C57BL/6 vs *Scnn1b*-Tg	0.0251
Fig 5C<br>CXCL10	Ordinary Two-way ANOVA	Interaction	0.5565	C57BL/6<br>Sterile vs PAO1 beads	0.0320
		Genotype	0.3524	*Scnn1b*-Tg<br>Sterile vs. PAO1 beads	0.1814
		Infection	0.0185	Sterile beads<br>C57BL/6 vs *Scnn1b*-Tg	0.3003
				PAO1 beads<br>C57BL/6 vs *Scnn1b*-Tg	0.7976
Fig 5D<br>KC/GRO	Ordinary Two-way ANOVA	Interaction	0.0260	C57BL/6<br>Sterile vs PAO1 beads	0.3100
		Genotype	0.0533	*Scnn1b*-Tg<br>Sterile vs. PAO1 beads	0.0005
		Infection	0.0014	Sterile beads<br>C57BL/6 vs *Scnn1b*-Tg	0.8017
				PAO1 beads<br>C57BL/6 vs *Scnn1b*-Tg	0.0046
Fig 5D<br>MIP-2	Ordinary Two-way ANOVA	Interaction	0.1106	C57BL/6<br>Sterile vs PAO1 beads	0.1592
		Genotype	0.0568	*Scnn1b*-Tg<br>Sterile vs. PAO1 beads	0.0020
		Infection	0.0020	Sterile beads<br>C57BL/6 vs *Scnn1b*-Tg	0.8037
				PAO1 beads<br>C57BL/6 vs *Scnn1b*-Tg	0.0152
Fig 5E<br>MIP-3α	Ordinary Two-way ANOVA	Interaction	0.4493	C57BL/6<br>Sterile vs PAO1 beads	0.0026
		Genotype	0.0067	*Scnn1b*-Tg<br>Sterile vs. PAO1 beads	0.0004
		Infection	<0.0001	Sterile beads<br>C57BL/6 vs *Scnn1b*-Tg	0.1244
				PAO1 beads<br>C57BL/6 vs *Scnn1b*-Tg	0.0119
Fig 5E<br>IL-15	Ordinary Two-way ANOVA	Interaction	0.6408	C57BL/6<br>Sterile vs PAO1 beads	0.0485
		Genotype	0.3487	*Scnn1b*-Tg<br>Sterile vs. PAO1 beads	0.1486
		Infection	0.0225	Sterile beads<br>C57BL/6 vs *Scnn1b*-Tg	0.3249
				PAO1 beads<br>C57BL/6 vs *Scnn1b*-Tg	0.7299
Fig 5E<br>IL-16	Ordinary Two-way ANOVA	Interaction	0.2681	C57BL/6<br>Sterile vs PAO1 beads	0.4489
		Genotype	0.2692	*Scnn1b*-Tg<br>Sterile vs. PAO1 beads	0.0353
		Infection	0.0425	Sterile beads<br>C57BL/6 vs *Scnn1b*-Tg	0.9985

				PAO1 beads<br>C57BL/6 vs *Scnn1b*-Tg	0.1147
Fig 6B<br>IL-2	Ordinary Two-way ANOVA	Interaction	0.1368	C57BL/6<br>Sterile vs PAO1 beads	0.0141
		Genotype	0.0111	*Scnn1b*-Tg<br>Sterile vs. PAO1 beads	0.0002
		Infection	<0.0001	Sterile beads<br>C57BL/6 vs *Scnn1b*-Tg	0.3781
				PAO1 beads<br>C57BL/6 vs *Scnn1b*-Tg	0.0053
Fig 6B<br>IL-27p28/IL-30	Ordinary Two-way ANOVA	Interaction	0.7477	C57BL/6<br>Sterile vs PAO1 beads	0.1430
		Genotype	0.0031	*Scnn1b*-Tg<br>Sterile vs. PAO1 beads	0.0634
		Infection	0.0244	Sterile beads<br>C57BL/6 vs *Scnn1b*-Tg	0.0475
				PAO1 beads<br>C57BL/6 vs *Scnn1b*-Tg	0.0112
Fig 6B<br>IFNγ	Ordinary Two-way ANOVA	Interaction	0.3031	C57BL/6<br>Sterile vs PAO1 beads	0.1949
		Genotype	0.2980	*Scnn1b*-Tg<br>Sterile vs. PAO1 beads	0.0150
		Infection	0.0104	Sterile beads<br>C57BL/6 vs *Scnn1b*-Tg	0.9936
				PAO1 beads<br>C57BL/6 vs *Scnn1b*-Tg	0.1389
Fig 6C<br>IL-4	Ordinary Two-way ANOVA	Interaction	0.0818	C57BL/6<br>Sterile vs PAO1 beads	0.6675
		Genotype	0.0055	*Scnn1b*-Tg<br>Sterile vs. PAO1 beads	0.0115
		Infection	0.0275	Sterile beads<br>C57BL/6 vs *Scnn1b*-Tg	0.0026
				PAO1 beads<br>C57BL/6 vs *Scnn1b*-Tg	0.3366
Fig 6C<br>IL-5	Ordinary Two-way ANOVA	Interaction	0.8484	C57BL/6<br>Sterile vs PAO1 beads	0.6781
		Genotype	0.0003	*Scnn1b*-Tg<br>Sterile vs. PAO1 beads	0.5323
		Infection	0.4599	Sterile beads<br>C57BL/6 vs *Scnn1b*-Tg	0.0039
				PAO1 beads<br>C57BL/6 vs *Scnn1b*-Tg	0.0067
Fig 6C<br>IL-33	Ordinary Two-way ANOVA	Interaction	0.1584	C57BL/6<br>Sterile vs PAO1 beads	0.0822
		Genotype	0.8066	*Scnn1b*-Tg<br>Sterile vs. PAO1 beads	0.0025
		Infection	0.0014	Sterile beads<br>C57BL/6 vs *Scnn1b*-Tg	0.3952

				PAO1 beads<br>C57BL/6 vs *Scnn1b*-Tg	0.2390
Fig 6D	Ordinary Two-way ANOVA	Interaction	0.0082	C57BL/6<br>Sterile vs PAO1 beads	0.0132
		Genotype	0.0046	*Scnn1b*-Tg<br>Sterile vs. PAO1 beads	<0.0001
		Infection	<0.0001	Sterile beads<br>C57BL/6 vs *Scnn1b*-Tg	0.8461
				PAO1 beads<br>C57BL/6 vs *Scnn1b*-Tg	0.0003
Fig 6F	Ordinary Two-way ANOVA	Interaction	0.9120	C57BL/6<br>Sterile vs PAO1 beads	0.0144
		Genotype	0.0019	*Scnn1b*-Tg<br>Sterile vs. PAO1 beads	0.0194
		Infection	0.0020	Sterile beads<br>C57BL/6 vs *Scnn1b*-Tg	0.0140
				PAO1 beads<br>C57BL/6 vs *Scnn1b*-Tg	0.0188
Fig S2A	Student t-test	-	-	C57BL/6 vs *Scnn1b*-Tg	0.0452
Fig S2B Resistance	Student t-test	-	-	C57BL/6 vs *Scnn1b*-Tg	0.8965
Fig S2B Elastance	Student t-test	-	-	C57BL/6 vs *Scnn1b*-Tg	0.0041
Fig S2C Airway resistance	Student t-test	-	-	C57BL/6 vs *Scnn1b*-Tg	0.6370
Fig S2C Tissue damping	Student t-test	-	-	C57BL/6 vs *Scnn1b*-Tg	0.0542
Fig S2C Tissue elastance	Student t-test	-	-	C57BL/6 vs *Scnn1b*-Tg	0.0026
Fig S2D Compliance	Student t-test	-	-	C57BL/6 vs *Scnn1b*-Tg	0.0002
Fig S2D Hysteresivity	Student t-test	-	-	C57BL/6 vs *Scnn1b*-Tg	0.0253
Fig S2F FVC	Student t-test	-	-	C57BL/6 vs *Scnn1b*-Tg	0.0011
Fig S2F FEV0.1	Student t-test	-	-	C57BL/6 vs *Scnn1b*-Tg	0.0136
Fig S2F FEV0.1/FVC	Mann-Whitney	-	-	C57BL/6 vs *Scnn1b*-Tg	0.0286
Fig S2F PEF	Student t-test	-	-	C57BL/6 vs *Scnn1b*-Tg	0.4336
Fig S2F FEF0.1	Student t-test	-	-	C57BL/6 vs *Scnn1b*-Tg	0.0001
Fig S3A	Student t-test	-	-	C57BL/6 vs *Scnn1b*-Tg	0.0049

Fig S3B	Student t-test	-	-	C57BL/6 vs *Scnn1b*-Tg	0.0119
Fig S3C	Student t-test	-	-	C57BL/6 vs *Scnn1b*-Tg	0.0301
Fig S3D	Student t-test	-	-	C57BL/6 vs *Scnn1b*-Tg	0.0449
Fig S3E	Student t-test	-	-	C57BL/6 vs *Scnn1b*-Tg	0.0034
Fig S3F	Student t-test	-	-	C57BL/6 vs *Scnn1b*-Tg	0.0044
Fig S3G	Student t-test	-	-	C57BL/6 vs *Scnn1b*-Tg	0.0027
Fig S3H	Student t-test	-	-	C57BL/6 vs *Scnn1b*-Tg	0.0341
Fig S4A	Student t-test	-	-	C57BL/6 vs *Scnn1b*-Tg	0.0245
Fig S4B	Student t-test	-	-	C57BL/6 vs *Scnn1b*-Tg	0.0133
Fig S4C	Student t-test	-	-	C57BL/6 vs *Scnn1b*-Tg	0.0997
Fig S4D Naïve	Student t-test	-	-	C57BL/6 vs *Scnn1b*-Tg	0.1929
Fig S4D Effector	Student t-test	-	-	C57BL/6 vs *Scnn1b*-Tg	0.0078
Fig S4D Central memory	Student t-test	-	-	C57BL/6 vs *Scnn1b*-Tg	0.0699
Fig S4E Naïve	Student t-test	-	-	C57BL/6 vs *Scnn1b*-Tg	0.2596
Fig S4E Effector	Student t-test	-	-	C57BL/6 vs *Scnn1b*-Tg	0.0128
Fig S4E Central memory	Mann-Whitney	-	-	C57BL/6 vs *Scnn1b*-Tg	0.6905
Fig S4F	Student t-test	-	-	C57BL/6 vs *Scnn1b*-Tg	0.2536
Fig S4G	Student t-test	-	-	C57BL/6 vs *Scnn1b*-Tg	0.0290

Table S2. Antibodies used for inflammatory flow cytometry panel.

Cell surface antibodies	Conjugation	Detection channel	Vendor, catalog #
LIVE/DEAD Yellow stain	-	V8	Invitrogen cat# L34967
CD170 (Siglec F)	BV421	V1	BD Biosciences cat# 562681
CD4	Pacific Blue	V3	Biolegend cat# 100427
CD45	BV510	V7	Biolegend cat# 103137
CD62L	BV605	V10	Biolegend cat# 104437
CD8a	BV650	V11	Biolegend cat# 100741
CD44	BV785	V15	Biolegend cat# 103041
CD11b	AF 532	B3	Invitrogen cat# 58011282
TCRb	PE-CF594	B6	BD Biosciences cat# 562841
CD64	PerCP-Cy5.5	B9	Biolegend cat# 139307
Ly6C	RB780	B14	BD Biosciences cat# 568739
CD11c	AF 700	R4	Biolegend cat# 117319
Ly6G	APC-Fire 750	R7	Biolegend cat# 127651
**Intracellular antibody**	**Conjugation**	**Detection channel**	**Vendor, catalog #**
Foxp3	PE	B4	Miltenyi Biotec cat# 130-111-678
